# Single‐Step Insertion of Organic Sulfur Into a Fe_6_C Carbide Carbonyl Cluster, Including the Natural Amino Acid *L*‐Cysteine: Vibrational Circular Dichroism and Chirality Transfer

**DOI:** 10.1002/anie.202513702

**Published:** 2025-08-01

**Authors:** Francesca Forti, Andrea Pellegrini, Cristiana Cesari, Cristina Femoni, Maria Carmela Iapalucci, Michele Mancinelli, Stefano Zacchini

**Affiliations:** ^1^ Dipartimento di Chimica Industriale “Toso Montanari” Università di Bologna Via P. Gobetti 85‐40129 Bologna Italy

**Keywords:** Chirality transfer, Cysteine, Iron carbide cluster, Molecular cluster, Vibrational circular dichroism

## Abstract

We present a straightforward and versatile synthetic route to Fe_6_C carbide carbonyl clusters containing organosulfur ligands, including the chiral amino acid *L*‐ and *D*‐cysteine. Reaction of the reduced cluster [NEt_4_]_4_[Fe_6_C(CO)_15_] (**1**) with thiols (RSH) or disulfides (RSSR) affords novel functionalized hexa‐iron carbide clusters [NEt_4_]_3_[Fe_6_C(CO)_14_(SR)] (R = CH_3_, **2**; C_6_H_5_, **3**; *p*‐C_6_H_4_CH_3_, **4**; *L*‐cysteine, ***L***‐**5**; *D*‐cysteine, ***D***‐**5**). Compounds **2–5** have been characterized by Fourier transform infrared (FT‐IR), ^1^H and ^13^C{^1^H} nuclear magnetic resonance (NMR) spectroscopy, and the molecular structures of **2–4** have been determined by single‐crystal X‐ray diffraction (SC‐XRD). The chirality of the ***L*‐5** and ***D*‐5** enantiomers has been investigated by vibrational circular dichroism (VCD), and this represents the first VCD study of chiral metal carbonyl clusters. Combined analyses of VCD and calculated density functional theory (DFT) spectra clearly point out the occurrence of chirality transfer from the chiral organosulfur ligand to the CO ligands, as indicated by the presence of VCD bands in the ν_CO_ region of both ***L*‐5** and ***D*‐5**. This unprecedented transfer of chirality among different ligands arises from close interactions between the CH_2_ and CH hydrogens of coordinated cysteine and the CO shell of the clusters.

## Graphical abstract

We describe a one‐pot synthetic procedure for the functionalization with organosulfur ligands of hexa‐iron carbide carbonyl clusters. This synthetic procedure is highly versatile and may be applied to alkyl, aryl, and functionalized organic sulfur reagents, including the chiral amino acids *L‐* and *D‐*cysteine. Chirality transfer from cysteine to the CO ligands of the Fe_6_C cluster has been observed by vibrational circular dichroism (VCD).

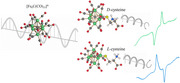

## Introduction

Nitrogenase enzymes play a crucial role in nature, since their unique ability to accomplish nitrogen fixation. The active site of the FeMoco cofactor of nitrogenase contains a Fe_6_C carbide cluster bonded to both inorganic and organic sulfur species, the former as sulfide ions and the latter in the form of the naturally occurring amino acid *L*‐cysteine.^[^ ^1^, ^2^, ^3^, ^4^, ^5^, ^6^ ^]^ The purely inorganic carbide is enclosed within a trigonal prismatic cage composed of Fe^2+^ and Fe^3+^ centers. Owing to the presence of weak‐field S‐based ligands, the resulting cluster is in a high spin state. The chemical synthesis of the FeMoco cofactor remains a significant challenge in the fields of biochemistry and bioinorganic chemistry.^[^ ^7^, ^8^, ^9^, ^10^ ^]^

Among the various strategies investigated for synthesizing model clusters of FeMoco,^[^ ^11^, ^12^, ^13^, ^14^, ^15^ ^]^ one possible route involves coordinating both inorganic and organic S‐based ligands to preformed Fe_6_C carbide carbonyl clusters.^[^ ^16^, ^17^, ^18^, ^19^ ^]^ The chemistry of these organometallic clusters, particularly [Fe_6_C(CO)_16_]^2−^, has been well known since the 1960s.^[^ ^20^, ^21^, ^22^, ^23^, ^24^, ^25^ ^]^ It might be remarked that [Fe_6_C(CO)_16_]^2−^ differs markedly compared to FeMoco. It possesses an octahedral rather than a trigonal prismatic cage, and the Fe centers display low oxidation states. Additionally, the strong‐field π‐acidic CO ligands render these organometallic clusters diamagnetic. Nevertheless, the stepwise replacement of high‐field carbonyl ligands with an increasing number of weak‐field S‐donors might reduce such differences, while preserving the Fe_6_C carbide core.

[Fe_6_C(CO)_16_]^2−^ is rather inert toward CO‐substitution and, only recently, suitable protocols have been developed for this purpose.^[^ ^26^, ^27^ ^]^ Its reaction with S_2_Cl_2_ results in the direct introduction of inorganic sulfur, affording a mixture of products: {[(CO)_15_(μ_6_‐C)Fe_6_]‐(μ_4_‐S)[Fe_5_(μ_5_‐C)(CO)_13_]}^2−^, [{Fe_4_(κ_2_S‐κ_4_C)(CO)_10_}(μ_3_‐S)(μ_3_‐S_2_)Fe(CO)_3_], and Fe_3_S_2_(CO)_9_.^[^ ^16^ ^]^ In the first product, the Fe_6_C cage is partially retained, whereas the second complex [{Fe_4_(κ_2_S‐κ_4_C)(CO)_10_}(μ_3_‐S)(μ_3_‐S_2_)Fe(CO)_3_] contains a {C‐S} motif that resembles the proposed biogenesis pathway for carbide insertion into the nitrogenase cofactor.^[^ ^28^, ^29^ ^]^ The reaction of [Fe_6_C(CO)_16_]^2−^ with S_8_ in the presence of [Fe_5_C(CO)_15_] results in a mixture of S‐containing clusters, including {[Fe_5_(μ_5_‐C)(CO)_13_]_2_(μ_4_‐S)}, [{Fe_4_(κ_2_S‐κ_4_C)(CO)_10_}(μ_3_‐S)_2_Fe(CO)_3_], and Fe_3_S_2_(CO)_9_, without retention of the Fe_6_C cage.^[^ ^16^ ^]^ Liu et al. developed a multistep synthesis of [Fe_6_C(CO)_14_(S)]^2−^, where the first step was CO‐substitution of [Fe_6_C(CO)_16_]^2−^ by SO_2_ affording [Fe_6_C(CO)_15_(SO_2_)]^2−^. This was followed by oxygen removal using CF_3_SO_3_CH_3_, BF_3_ and Cp_2_Co (or NaC_10_H_8_; Cp = C_5_H_5_) in sequence.^[^ ^19^ ^]^ The chemistry of [Fe_6_C(CO)_14_(S)]^2−^ has been further investigated, including redox reactions, substitutions, and metalations.^[^ ^18^ ^]^

Organic sulfur has been introduced into Fe carbide carbonyl clusters in only one reported case.^[^ ^16^ ^]^ The reaction of [Fe_6_C(CO)_16_]^2−^ with *p*‐Me‐C_6_H_4_‐SCl (generated in situ) afforded [Fe_5_C(CO)_13_(S‐C_6_H_4_Me)]^−^, with partial degradation of the Fe_6_C cage to Fe_5_C, likely due to the electrophilic nature of the organosulfur reagent.

The introduction of other organic ligands into the coordination sphere of [Fe_6_C(CO)_16_]^2−^ is not straightforward, especially when retention of Fe_6_C cage is desired. Cobb et al. achieved CO to phosphine substitution upon oxidation of [Fe_6_C(CO)_16_]^2−^ via an unsaturated [Fe_6_C(CO)_16_] species.^[^ ^26^ ^]^ However, this led predominantly to pentanuclear species such as [HFe_5_C(CO)_13_(PPh_3_)]^−^, [Fe_5_C(CO)_14_(PPh_3_)], and [Fe_5_C(CO)_14_(Triphos)] (Triphos = PhP(CH_2_CH_2_PPh_2_)_2_). Recently, our group demonstrated that starting from the more reduced [Fe_6_C(CO)_15_]^4−^,^[^ ^30^ ^]^ and performing its oxidation in the presence of phosphine ligands enables the synthesis of heteroleptic CO/PR_3_ clusters with retention of the Fe_6_C cage, as in the case of [Fe_6_C(CO)_15_(PTA)]^2−^ (PTA = 1,3,5‐triaza‐7‐phosphaadamantane).^[^ ^27^ ^]^ This procedure may be extended to harder ligands, such as CO_3_^2−^, and [Fe_6_C(CO)_14_(CO_3_)]^4−^ has been obtained upon oxidation of [Fe_6_C(CO)_15_]^4−^ in the presence of a base.

In this paper, we present a further extension of CO substitution with organosulfur ligands enabled by oxidation of [Fe_6_C(CO)_15_]^4−^. Specifically, the reaction of [NEt_4_]_4_[Fe_6_C(CO)_15_] (**1**) with thiols (RSH) or disulfides (RSSR) affords the first examples of Fe_6_C carbide carbonyl clusters containing organic sulfur ligands: [NEt_4_]_3_[Fe_6_C(CO)_14_(SR)] (R = CH_3_, **2**; C_6_H_5_, **3**; *p*‐C_6_H_4_CH_3_, **4**; *L*‐cysteine, ***L***‐**5**; *D*‐cysteine, ***D***‐**5**). Notably, this approach allowed the direct coordination of the natural amino acid *L*‐cysteine, as well as its enantiomer *D*‐cysteine. Both cluster enantiomers, ***L*‐5** and ***D*‐5**, have been characterized by vibrational circular dichroism (VCD). This represents the first VCD study of chiral metal carbonyl clusters, and chirality transfer from the chiral organic ligand to the overall conformation of the CO ligands has been unequivocally demonstrated.

VCD is a powerful tool for studying the chiral properties of molecules, providing insight into their absolute configuration and sensitivity to conformational structures, making it particularly well‐suited for the study of chiral systems.^[^ ^31^, ^32^, ^33^, ^34^ ^]^ Traditionally, VCD has been widely applied for the study of chirality in small organic molecules,^[^ ^35^, ^36^ ^]^ natural products,^[^ ^37^, ^38^ ^]^ proteins,^[^ ^39^ ^]^ and coordination complexes.^[^ ^40^, ^41^, ^42^, ^43^ ^]^

Recently, VCD has also been employed to investigate chirality in Au and Ag ligand‐protected molecular nanoclusters.^[^ ^44^, ^45^, ^46^, ^47^, ^48^ ^]^ These studies have focused on both chirality induced by enantiomerically pure ligands, and chirality transfer from a chiral metal core to a shell of achiral organic ligands. In this work, we demonstrate for the first time that chirality can be transferred from a single chiral organic ligand to CO ligands bonded to an organometallic cluster.

## Results and Discussion

### Synthesis of [NEt_4_]_3_[Fe_6_C(CO)_14_(SR)]

**Scheme 1:**
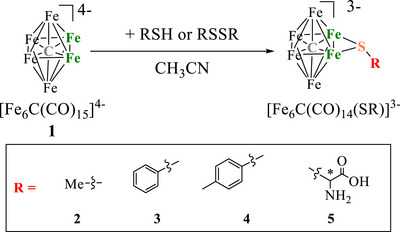
Synthesis of **2–5**. CO ligands have been omitted for clarity. [NEt_4_]^+^ cations have been employed for all anions. *Both enantiomers *L*‐cysteine and *D*‐cysteine have been employed leading to enantiopure products ***L*‐5** and ***D*‐5**.

The addition of thiols (RSH) or disulfides (RSSR) to a CH_3_CN solution of [NEt_4_]_4_[Fe_6_C(CO)_15_] (**1**) affords the new species with the general formula [NEt_4_]_3_[Fe_6_C(CO)_14_(SR)] (R = CH_3_, **2**; C_6_H_5_, **3**; *p*‐C_6_H_4_CH_3_, **4**; *L*‐cysteine, ***L***‐**5**; *D*‐cysteine, ***D***‐**5**) (Scheme 1). The choice between thiols or disulfides as reagents mainly depends on their commercial availability. When both are available for the same R‐group, as in the case of PhSH and PhSSPh (Ph = C_6_H_5_), the same final product has been obtained in comparable yields. The stoichiometry of the reaction strongly depends on the nature of the reagent. The reagent must be added slowly and in small portions to the solution of **1**, while monitoring the reaction by Fourier Transform Infrared (FT‐IR) spectroscopy, to avoid decomposition. In all cases, the formation of compounds **2–5** is accompanied by the appearance of oxidation products such as [NEt_4_]_2_[Fe_6_C(CO)_16_] (**6**), [NEt_4_]_2_[Fe_5_C(CO)_14_] (**7**), and [NEt_4_]_2_[Fe_4_C(CO)_12_] (**8**). These oxidized products can be extracted in tetrahydrofuran (THF) or acetone, whereas **2–5** are soluble in CH_3_CN. This procedure is rather versatile, allowing the introduction of various RS‐groups, including aliphatic and aromatic groups, as well as functionalized moieties, such as *L*‐ and *D‐*cysteine. All the new species **2–5**, as well as the side products **6–8**,^[^ ^20^, ^49^, ^50^ ^]^ have been identified by FT‐IR spectroscopy (Figures S1–S10 in the Supporting Information). Moreover, crystals suitable for single crystal X‐ray diffraction (SC‐XRD) analysis have been obtained in the case of **2–4**.^[^ ^51^ ^]^

The FT‐IR spectra of **2–5** in CH_3_CN solution display carbonyl stretching (ν_CO_) bands at 1907–1918 and 1720–1734 cm^−1^ corresponding to terminal and edge bridging carbonyls, respectively. These compounds absorb at intermediate values between those of **1** (*ν*_CO_ = 1875 and 1688 cm^−1^) and **6** (*ν*_CO_ = 1966 and 1772 cm^−1^), in agreement with their 3– charge, as also found in related Fe_6_C^[^ ^27^ ^]^ and Ru_6_C clusters.^[^ ^52^ ^]^

Clusters **2–5**, as well as the parent **1** and closely related **6**, are diamagnetic species which are electron precise octahedral clusters possessing 86 cluster valence electrons (CVE).^[^ ^26^, ^27^, ^30^ ^]^ According to organometallic electron‐counting rules,^[^ ^53^ ^]^ the interstitial carbide is assumed as a neutral ligand donating four valence electrons. Each Fe atom contributes with eight electrons and each CO ligand with two electrons, whereas μ‐SR ligand contributes with three electrons. Then, the negative charge must be added. Thus, **1** possesses overall 6 × 8 (6 Fe) + 1 × 4 (1 C) + 15 × 2 (15 CO) + 4 (negative charge) = 86 CVE, **2‐5** have 6 × 8 (6 Fe) + 1 × 4 (1 C) + 14 × 2 (14 CO) + 1 × 3 (1 μ‐SR) + 3 (negative charge) = 86 CVE, and the electron count of **6** is 6 × 8 (6 Fe) + 1 × 4 (1 C) + 16 × 2 (16 CO) + 2 (negative charge) = 86 CVE.

In the ^13^C{^1^H} NMR spectra in CD_3_CN solution (Figures S11–S18 in the Supporting Information), the CO ligands of **2–5** appear as a singlet at *δ*_C_ = 236–238 ppm, suggesting a rapid scrambling of the carbonyls in solution. Similar behavior has been observed for **1** (singlet, *δ*_C_ = 244.6 ppm) and **6** (singlet, *δ*_C_ = 228 ppm). The carbide ligand resonates at *δ*_C_ = 488.9 ppm for **4**.

**Scheme 2:**
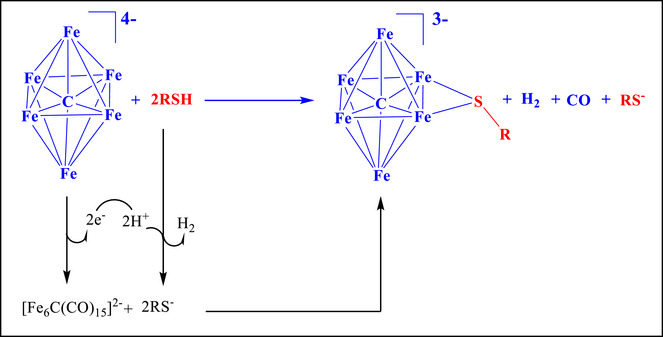
Proposed mechanism for the formation of the clusters **2–5** via oxidation and addition of an RS‐group to **1** using thiols (RSH).

**Scheme 3:**
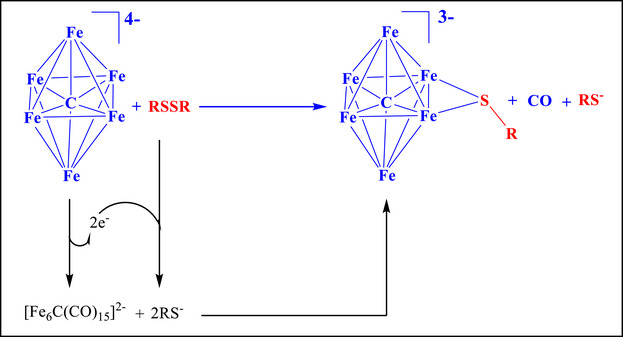
Proposed mechanism for the formation of the clusters **2–5** via oxidation and addition of an RS‐group to **1** using disulfides (RSSR).

Formation of **2–5** may be viewed as a two‐electron oxidation of **1** to generate an unsaturated [Fe_6_C(CO)_15_]^2−^ intermediate, followed by nucleophilic addition of RS^−^ and elimination of one CO ligand. The oxidants are H^+^ ions in the case of thiols RSH (Scheme 2), or the S─S bond in the case of disulfides RSSR (Scheme 3). In particular, when using RSH, oxidation of **1** occurs with the concomitant reduction of H^+^ (generated from RSH) to H_2_ (Scheme 2). In the case of disulfides, the two‐electron oxidation of **1** is accompanied by the reduction of RSSR to yield two RS^−^ ions (Scheme 3). The two‐electron oxidation of **1** with formation of **2–5** reduces π‐back‐donation to CO ligands and, as a direct consequence, the *ν*_CO_ bands of **2–5** are observed at higher wave‐numbers in the FT‐IR spectra compared to **1**.

The oxidation of **1** has recently been investigated both chemically and electrochemically, confirming the involvement of an unsaturated [Fe_6_C(CO)_15_]^2−^ species in the oxidative addition with ligands such as PR_3_ and CO_3_^2−^.^[^ ^27^ ^]^ Moreover, the unsaturated [Fe_6_C(CO)_15_]^2−^ species can add CO resulting in compound **6**, a side‐product often obtained along with **2–5**. The necessary CO can be obtained directly from the synthesis of **2–5**, since they contain 14 CO ligands, whereas 15 CO ligands are present in parent **1**; further CO may be also produced by partial decomposition processes.

### Molecular Structures of [NEt_4_]_3_[Fe_6_C(CO)_14_(SR)] (R = CH_3_, C_6_H_5_, and *p*‐C_6_H_4_CH_3_)

**Figure 1:**
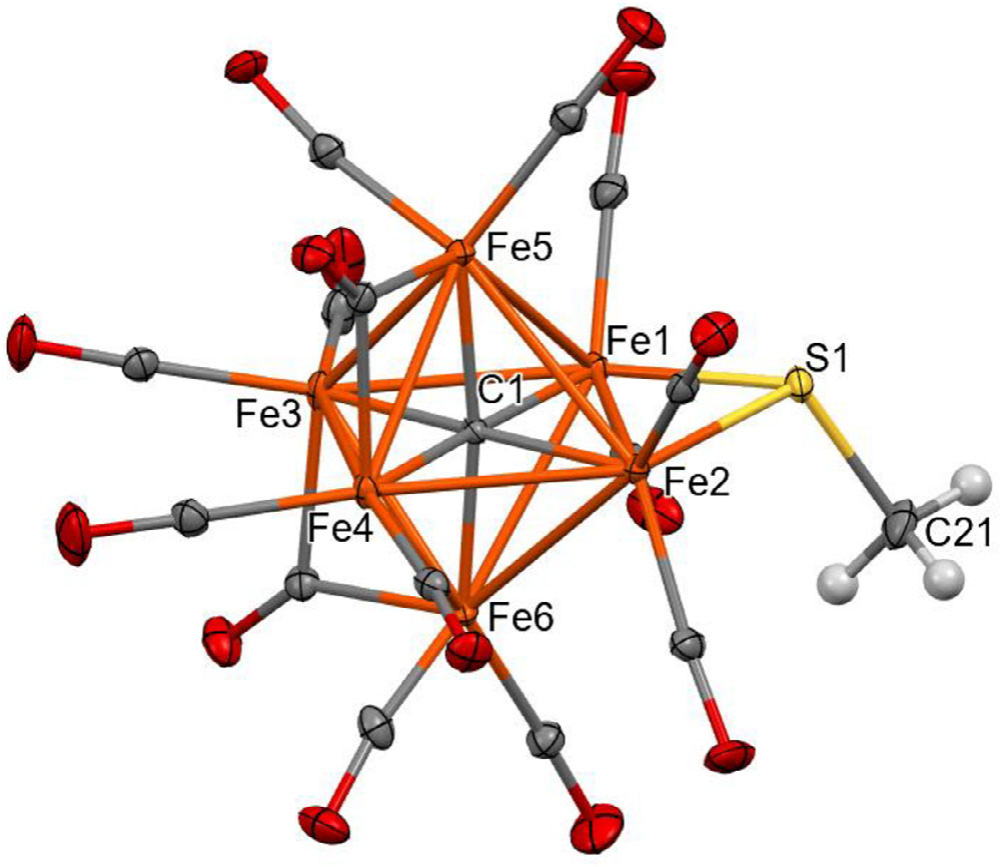
Molecular structure of **2** (orange, Fe; yellow, S; red, O; grey, C; and white, H). Thermal ellipsoids are shown at the 30% probability level. SC‐XRD data were collected at 100 K.

**Figure 2:**
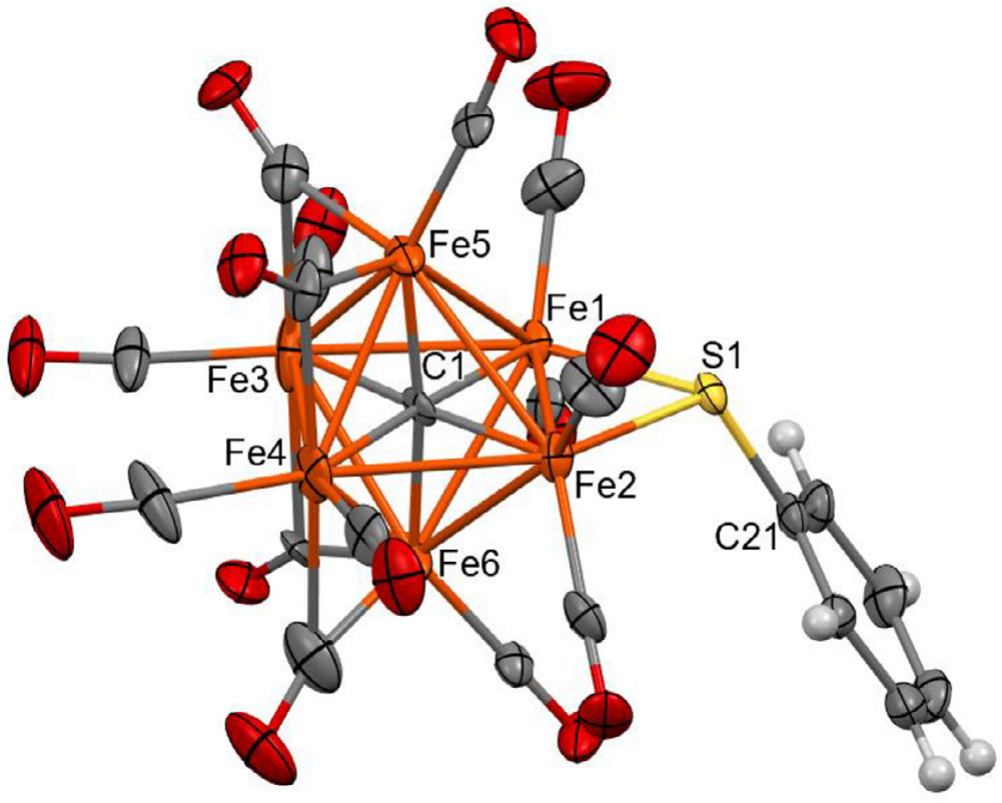
Molecular structure of **3** (orange, Fe; yellow, S; red, O; grey, C; and white, H). Thermal ellipsoids are shown at the 30% probability level. SC‐XRD data were collected at 100 K.

**Figure 3:**
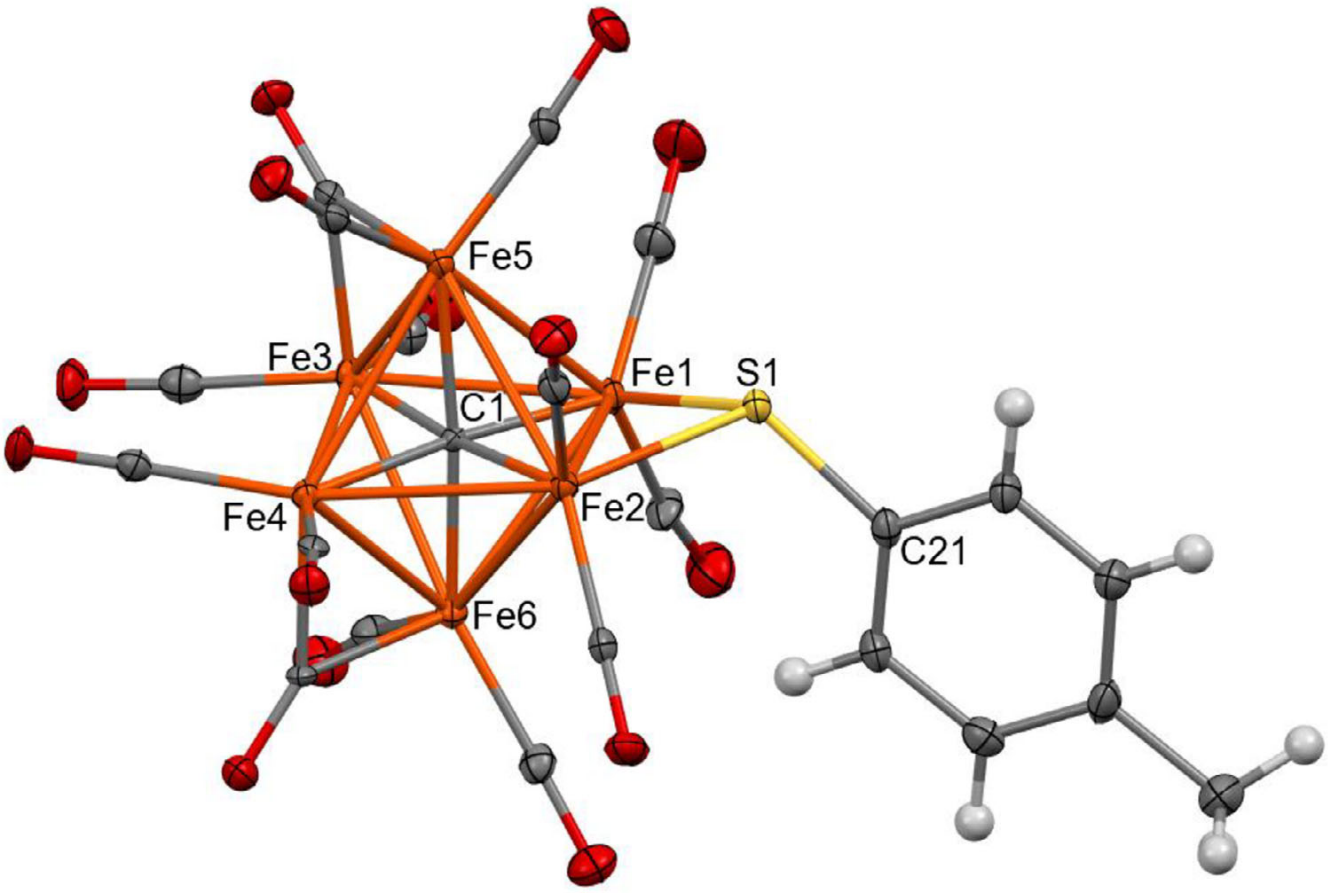
Molecular structure of **4** (orange, Fe; yellow, S; red, O; grey, C; and white, H). Thermal ellipsoids are shown at the 30% probability level. SC‐XRD data were collected at 100 K.

Clusters **2–4** feature an octahedral Fe_6_C core containing a fully interstitial μ_6_‐C carbide and an edge‐bridging μ‐SR thiolate ligand (Figures 1, 2, 3). According to SC‐XRD data collected at 100 K, all the species display 10 terminal carbonyl ligands. In the case of **3**, the remaining 4 CO ligands display bridge asymmetry parameters (α) 0.219(14), 0.226(12), 0.262(16), and 0.365(16) which fall within the typical range for semibridging carbonyls [0.1–0.6] (Table S3 in the Supporting Information).^[^ ^54^ ^]^ In contrast, in cluster **2** [*α* = 0.040(2), 0.078(3), 0.428(4), and 0.446(4)] and **4** [*α* = 0.006(4), 0.034(4), 0.548(6), and 0.688(7)], two CO ligands are perfectly edge‐bridging [*α* < 0.1], whereas the other two carbonyls are very close to be terminally bonded [*α* > 0.6]. Notably, collecting the SC‐XRD data of **2** at 293 K (Figure S28 in the Supporting Information) the stereochemistry of the CO ligands slightly changes and all four carbonyls may be described as semibridging [*α* = 0.137(6), 0.179(6), 0.326(8), and 0.356(7)] as in the case of the low temperature structure of **3**. Overall, the stereochemistry of these four CO ligands appears to be slightly affected by both the nature of the R‐group and temperature. This is also in keeping with the fact that the CO ligands are fluxional in solution, and a single resonance is present at room temperature in the ^13^C{^1^H} NMR spectra of **2–4**. At this regard, some care must be taken when comparing the CO stereochemistry in solution with that in the solid state. Indeed, in SC‐XRD studies, the structures are time averaged over all the molecules of the crystals and over the duration of the X‐ray experiment.

The Fe‐C_carbide_ distances are comprised in a very narrow range [1.873(3)–1.890(3) Å, average 1.881(7) Å for **2**; 1.840(12)–1.902(12) Å, average 1.87(3) Å for **3**; 1.875(5)–1.889(5) Å, average 1.881(12) Å for **4**] (Tables S3 and S4 in the Supporting Information), with values very similar to those observed in other Fe_6_C carbide carbonyl clusters.^[^ ^20^, ^27^, ^30^, ^55^ ^]^ These Fe–C_carbide_ distances are sensibly shorter than those found in FeMoco [2.00(2) Å],^[^ ^56^ ^]^ consistent with the adoption of an octahedral Fe_6_C structure in the carbonyl clusters described here, as opposed to the larger trigonal prismatic cavity found in FeMoco.

The Fe‐S distances [2.2130(9) and 2.2016(10) Å for **2**; 2.215(3) and 2.216(3) Å for **3**; 2.2216(19) and 2.2048(18) Å for **4**] are similar to those reported by Joseph et al. for [NEt_4_][Fe_5_C(CO)_13_(S‐*p*‐Tol)] [2.205(9) and 2.209(7) Å].^[^ ^16^ ^]^ The Fe‐S(cysteine) distance found in the FeMoco cofactor is 2.27 Å,^[^ ^56^ ^]^ but in this case the *L*‐cysteine is terminally bonded to a single Fe center.

The density functional theory (DFT) optimized structures of **2**–**4** (Figures S19–S21 in the Supporting Information) are in good agreement with the SC‐XRD data with root mean square deviation (RMSD) values ranging from 0.25 and 0.40 Å (calculated employing two different basis sets for comparison and validation of the computational protocol; Table S1 in the Supporting Information). The simulations confirm the μ‐S coordination mode of ‐SR ligands. The best superimpositions of the experimental SC‐XRD structures and the corresponding computed structures for **2**–**4** are shown in Figures S22 and S23 in the Supporting Information.

### VCD Studies of [NEt_4_]_3_[Fe_6_C(CO)_14_(*L*‐Cys)] and [NEt_4_]_3_[Fe_6_C(CO)_14_(*D*‐Cys)]

The enantiomers [NEt_4_]_3_[Fe_6_C(CO)_14_(*L*‐Cys)] (***L*‐5**) and [NEt_4_]_3_[Fe_6_C(CO)_14_(*D*‐Cys)] (***D*‐5**) were obtained by the direct reaction of **1** with *L*‐cysteine and *D*‐cysteine, respectively, as described above. The nature of these two enantiomers in solution was first confirmed by FT‐IR and NMR spectroscopy (Figures S10, S17 and S18 in the Supporting Information). Specifically, ***L*‐5** and ***D*‐5** display characteristic *ν*_CO_ bands at 1918(s) and 1734(w) cm^−1^ in the FT‐IR spectrum in CH_3_CN solution, and a singlet at *δ*_C_ = 238.5 ppm in the ^13^C{^1^H} NMR spectrum in CD_3_CN solution, consistent with the spectral features observed for the other [NEt_4_]_3_[Fe_6_C(CO)_14_(SR)] clusters herein described.

The chiral nature of ***L*‐5** and ***D*‐5** was further investigated by VCD spectroscopy. In order to limit decomposition during the VCD experiments, a substoichiometric amount of cysteine was added to **1**. Blank tests conducted on un‐substituted clusters **1** and **6–7** displayed negligible VCD signals, indicating the absence of chirality in these compounds (Figures S25–S27 in the Supporting Information). Likewise, analysis of pure *L*‐ and *D*‐cysteine, conducted in the same experimental conditions, showed negligible signals both in FT‐IR and VCD spectra in the CO stretching region of the clusters considered, ensuring a clear spectral window between 2200 and 1600 cm^−1^.

**Figure 4:**
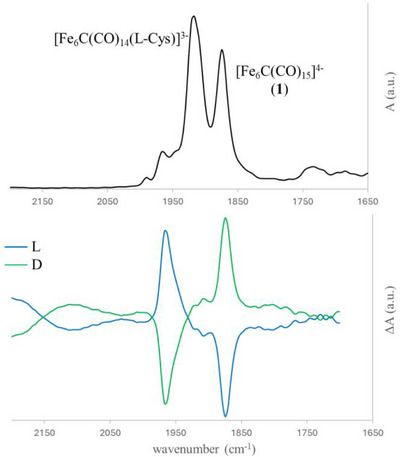
Experimental FT‐IR (top) and VCD (bottom) spectra of compounds ***L*‐5** and ***D*‐5** in CH_3_CN. The IR spectrum of *L*‐enantiomer is shown as representative example. The analysis was performed using a substoichiometric amount of cysteine, to avoid further oxidation reactions and, therefore, the residual band corresponding to the precursor **1** is still visible. VCD spectra of both ***L*‐5** (blue) and ***D*‐5** (green) enantiomers, resulting from independent experiments under the same conditions, are shown. IR and VCD were normalized to 1 a.u.

Notably, when the compounds ***L*‐5** and ***D*‐5** were tested, clear VCD signals were observed in the *ν*_CO_ region corresponding to the carbonyl stretching of the terminal CO ligands (Figure 4). Both enantiomers have been independently synthesized and analyzed, employing *L*‐cysteine and *D*‐cysteine as ligands, respectively. As expected, the VCD spectra of ***L*‐5** and ***D*‐5** are nonsuperimposable mirror images of each other, displaying signals of equal intensity but opposite sign, thus confirming their enantiomeric relationship (Figure 4).

To support the experimental results obtained, simulated VCD spectra have been calculated, starting from the DFT‐optimized structure of ***L*‐5**. A molecular dynamic simulation was performed on this structure to obtain eight snapshots, which have been subsequently optimized into eight distinct conformers (Gibbs free energy and Boltzmann populations are reported in Table S2 in the Supporting Information).

**Figure 5:**
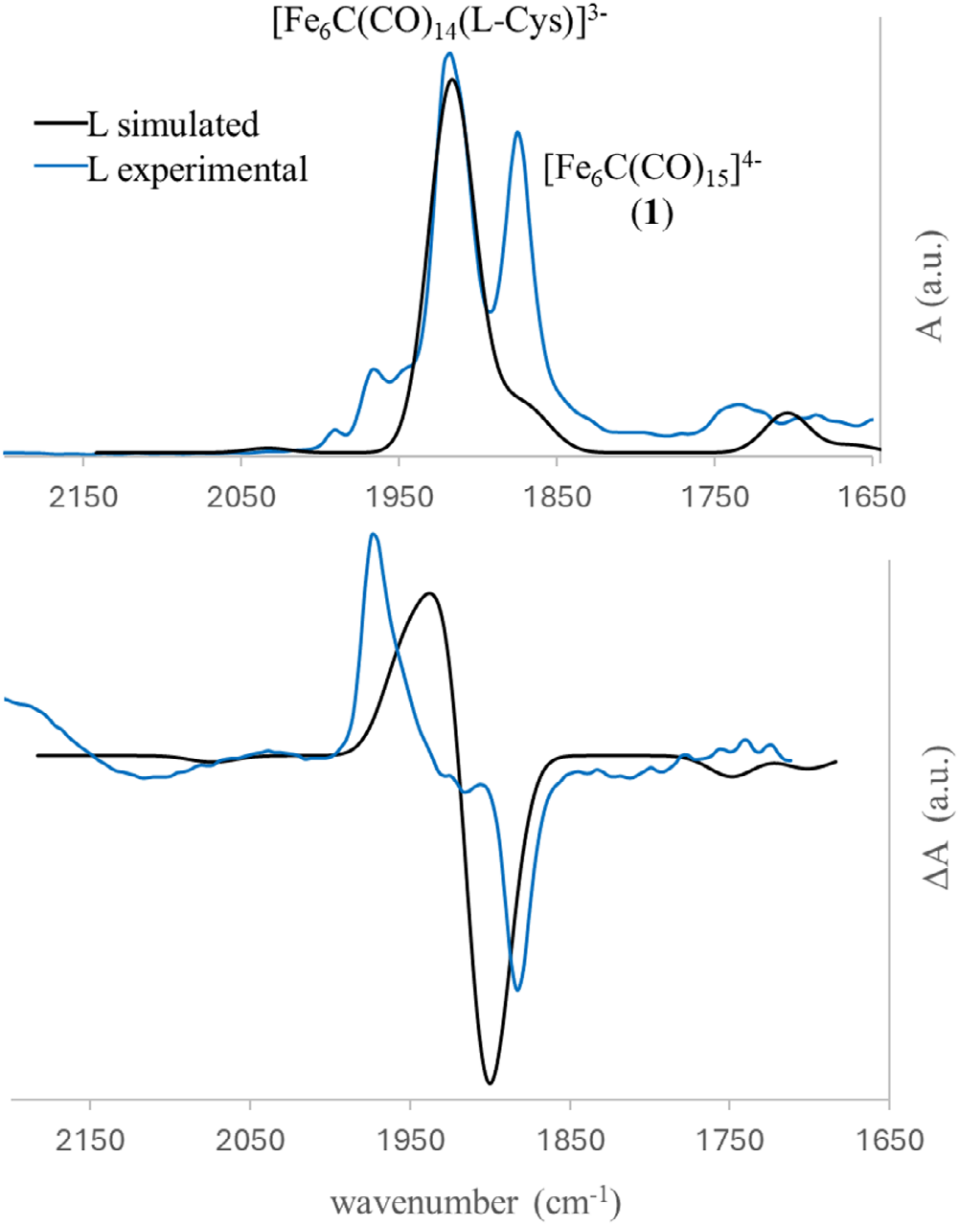
Experimental and simulated FT‐IR (top) and VCD (bottom) spectra of compound ***L*‐5**. Both the computed (black) and experimental (blue) spectra of ***L*‐5** are shown. The experimental analysis was conducted using a substoichiometric amount of *L*‐cysteine, to avoid further oxidation reactions, therefore the residual band corresponding to the precursor **1** is still visible in the FT‐IR spectrum. FT‐IR and VCD were normalized to 1 a.u.

The simulated VCD spectrum of each conformer was weighted according to its Boltzmann population and convoluted using a Gaussian broadening function with a full width at half maximum (FWHM) at 20 cm^−1^. To achieve the best agreement with the experimental VCD spectrum, a scaling factor of 0.98 had been applied; this same factor was also used for the simulated FT‐IR spectrum. The results of these calculations are shown in Figure 5, where the simulated VCD and FT‐IR spectra of the ***L*‐5** enantiomer are compared with the corresponding experimental spectra.

### Computational Studies of *L*‐5

**Figure 6:**
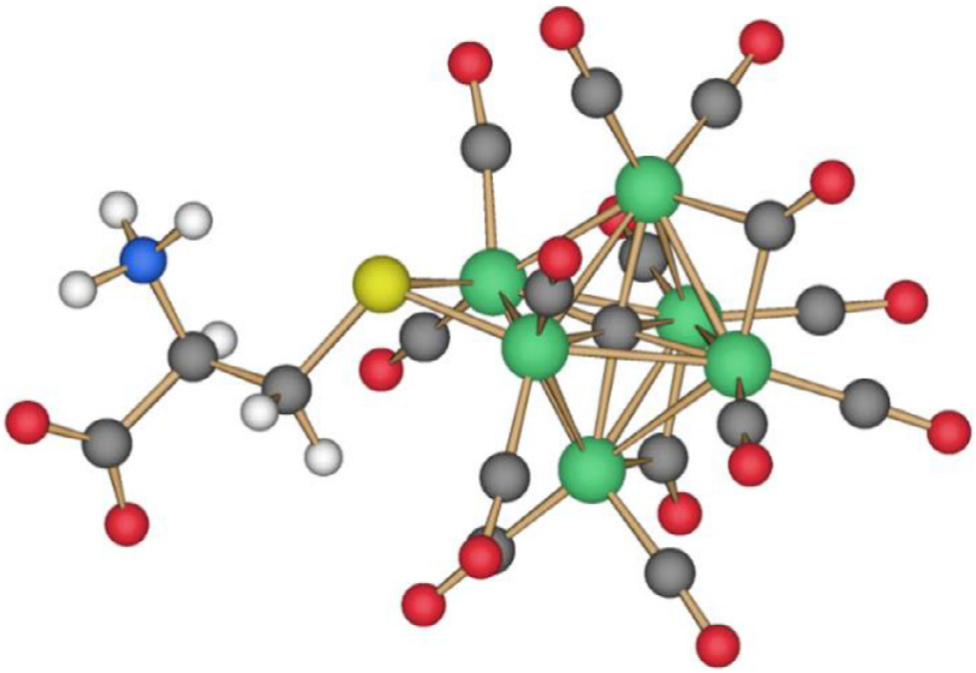
DFT‐optimized structure of ***L*‐5** (green Fe; red O; grey C; yellow S; white H; and blue N).

The molecular structure of ***L*‐5** has been simulated and optimized using DFT calculations (computational details can be found in the Supporting Information). The same computational methodology was applied to calculate the DFT‐optimized molecular structures of **2**–**4**, which all showed good agreement with experimental SC‐XRD data. This consistency validates the reliability of the computational approach for the systems herein described. The ground‐state geometry of ***L*‐5** (Figure 6) is composed of a Fe_6_C octahedron bearing the ligand *L*‐cysteine in a μ‐S bridging fashion. The coordination sphere of the metal core is completed by 12 terminal and 2 edge‐bridging CO ligands.

**Figure 7:**
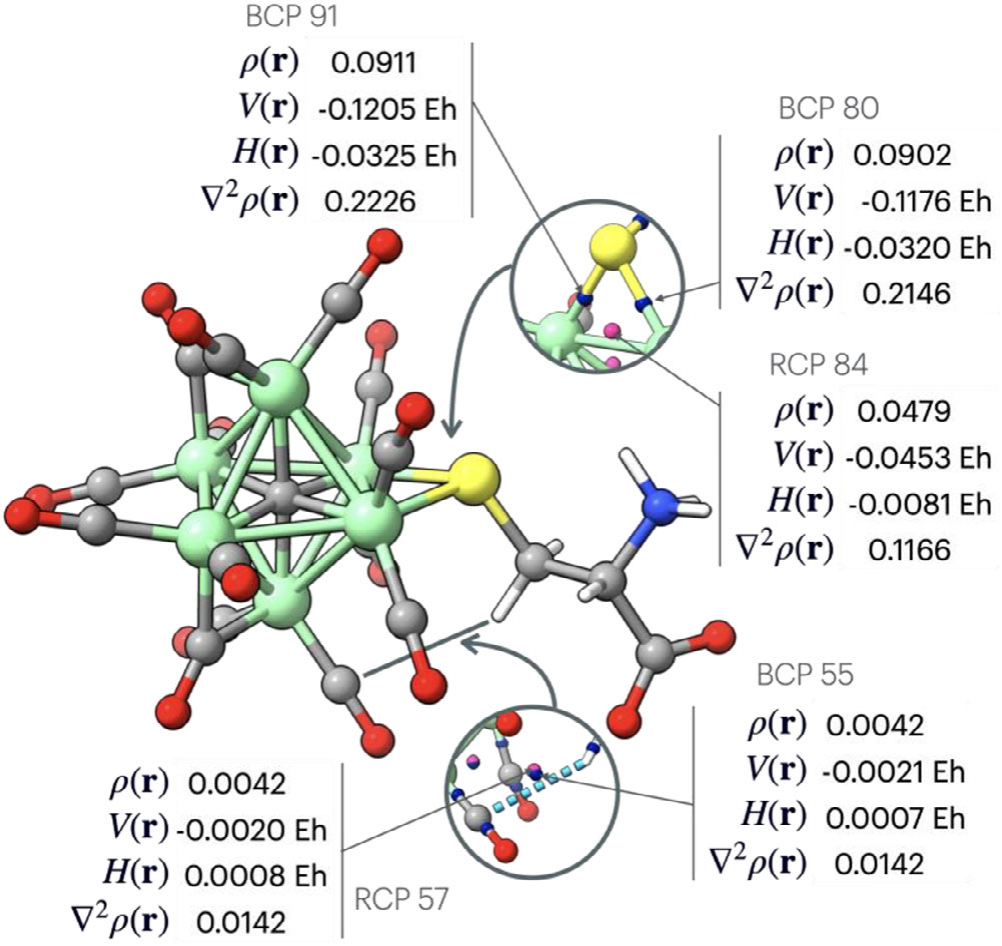
Selected critical points of the DFT optimized structure of ***L*‐5**: (3,−1) BCP represented in blue; (3,+1) RCP represented in pink. Values of electronic density ρ(r), potential electronic density *V*(r), total energy density *H*(r) and Laplacian of electronic density ∇^2^ρ(r) calculated at the selected critical points are reported.

A qualitative atom‐in‐molecule (AIM) analysis was performed on the lowest energy conformer of compound ***L*‐5** to further confirm the nature of the *L*‐cysteine coordination to the Fe_6_C core of the cluster. Critical points had been calculated from the Laplacian electron density; both classifications have been considered, the (3,−1) bond critical point (BCP) and the (3,+1) ring critical point (RCP), represented in Figure 7. A (3,−1) BCP is present on each of the two Fe‐S contacts between the μ‐S(cysteine) ligand and two Fe atoms of the cluster. Based on the computed properties at these two BCP's, the two Fe‐S interactions may be classified as dative bonds in agreement with Bianchi's definition.^[^ ^57^ ^]^ The presence of both a BCP and a RCP between one H‐atom of the cysteine methylene group and a CO ligand suggests a direct interaction between the chiral ligand and the carbonyl shell of the cluster. From the data obtained, it is qualitatively possible to estimate a moderate localization of the electron density in both the Fe─S bonds (BCP 80 and 91) with absence of electrons clustering along the bonds since the Laplacian of the density is positive. This suggests a close‐shell interaction and thus a dative bond with partial delocalization of the charge. RCP 84, instead, is located between two iron atoms and, due to the delocalization of the Fe─S bond, the density is lower but the interaction is still attractive. The hydrogen bond described both from the BCP 55 and the RCP 57 is characterized by a low‐density value and a small attractive interaction.

**Figure 8:**
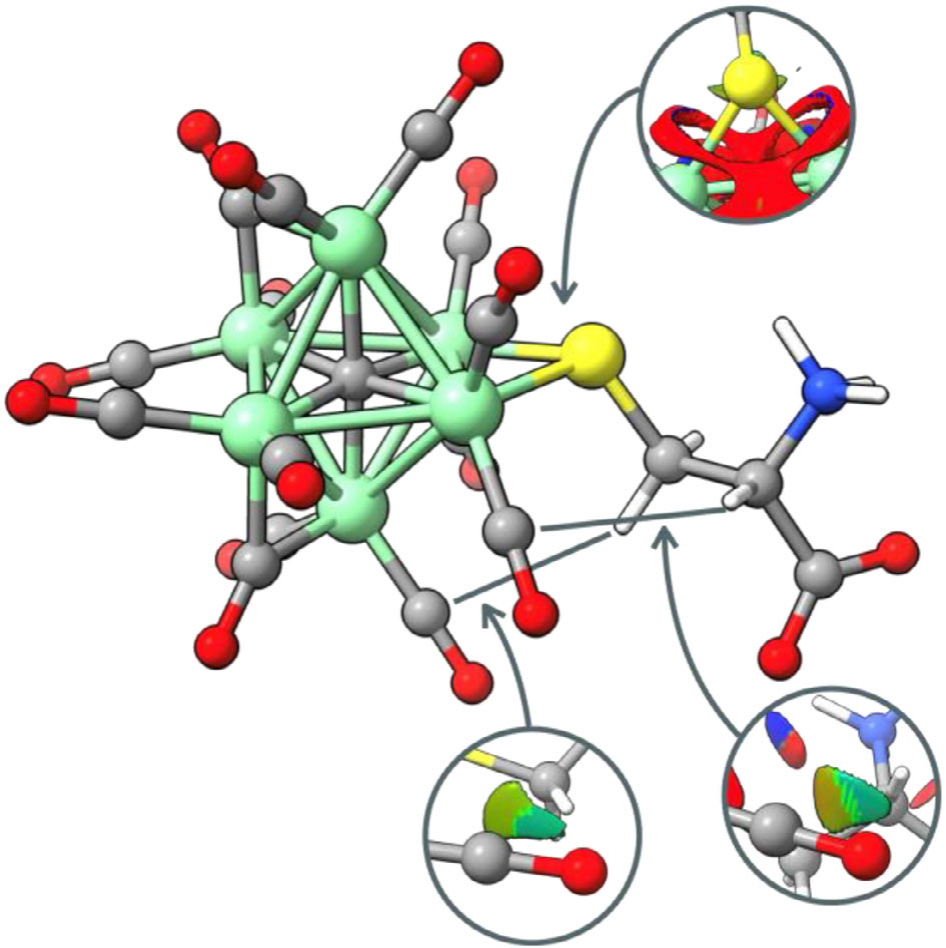
Relevant NCI surfaces highlighted of computed ***L*‐5**. Red color indicates strongly repulsive interactions, blue color indicates strongly attractive interaction, and green color corresponds to van der Waals coordination type.

To complete the qualitative analysis, Non‐Covalent Interaction (NCI) index surfaces were also investigated. The relevant surfaces for the computed compound ***L*‐5** are shown in Figure 8. In the drawing, the nature of the interactions follows a color‐coded representation: red indicates strongly repulsive interactions, blue indicates strongly attractive interaction, and green corresponds to van der Waals coordination type. NCI analysis highlights the presence of van der Waals interactions between the CH_2_ and CH hydrogens of the cysteine ligand and some CO ligands of the cluster. The interactions between the chiral organic ligand and the CO ligands depicted in Figures 7 and 8, and in particular that involving directly the chiral CH center, are likely to be responsible of the chirality transfer from the cysteine ligand to the CO ligands experimentally observed by VCD.

**Figure 9:**
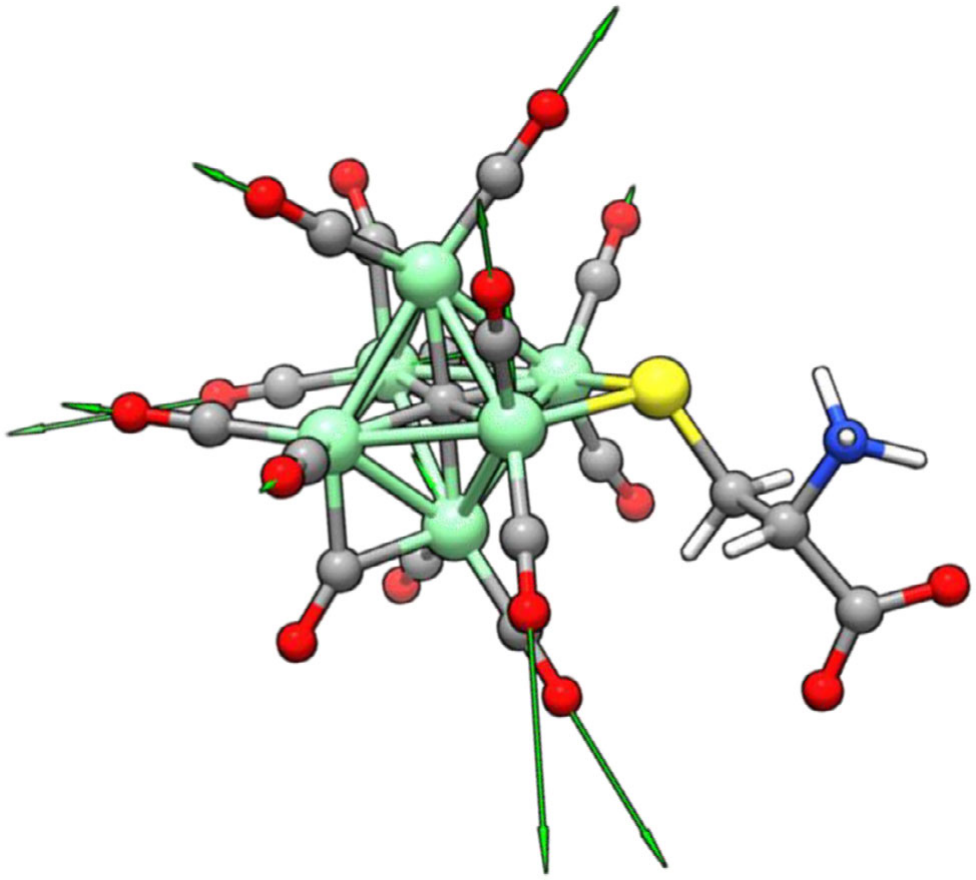
Displacement vectors of the most intense band of the simulated VCD spectrum of ***L*‐5**.

Indeed, based on the simulated structure of compound ***L*‐5**, the VCD transitions could be assigned to the carbonyl stretching (both symmetric and asymmetric). In particular, the CO vibrations corresponding to the most intense VCD band had been considered. Visualization of the normal mode displacement vectors (Figure 9) reveals that the two CO groups mostly involved in these vibrations are also those mostly interacting with the chiral moiety of the cysteine, as confirmed by the BCP points and the NCI surfaces of Figures 7 and 8.

## Conclusions

New hexa‐iron carbide carbonyl clusters functionalized with organosulfur ligands have been obtained by direct insertion of a RS‐group into a preformed Fe_6_C cluster. In particular, the highly reduced homoleptic cluster **1** reacts with thiols and disulfides affording the heteroleptic CO/SR clusters **2–5** with complete retention of the Fe_6_C cage. This synthetic procedure is highly versatile and may be applied to alkyl, aryl, and functionalized organic sulfur reagents, including the chiral amino acids *L‐* and *D‐*cysteine.

From a mechanistic point of view, the insertion of organic sulfur proceeds via a two‐electron oxidation of **1** involving a very reactive [Fe_6_C(CO)_15_]^2−^ unsaturated species. This intermediate rapidly undergoes addition of a RS^−^ moiety and elimination of a CO ligand, yielding the final products **2–5**. This represents a further extension to organosulfur ligands of the CO substitution enabled by oxidation of **1**, previously applied to other organic and inorganic ligands.^[^ ^27^ ^]^ The versatility of such synthetic route is herein further expanded to nitrogenase‐related metal cluster chemistry. Overall, the Fe_6_C iron‐carbide core may be viewed as a stable building block for the preparation of functionalized clusters. Becaause of the presence of semifilled Fe d orbitals, the contribution of the interstitial carbide and the interaction with surface ligands, Fe_6_C carbonyl clusters represents intriguing species for computational and theoretical investigation within the framework of the superatom model.^[^ ^58^ ^]^

The addition of *L‐* and *D‐*cysteine to **1** allowed, for the first time, the single‐step insertion of a chiral organosulfur ligand into the coordination sphere of an intact Fe_6_C carbide carbonyl cluster. The two enantiomers ***L*‐5** and ***D*‐5** display opposite VCD spectra in the *ν*_CO_ region, corroborating their chiral nature and enantiomeric relationship. This represents the first VCD study of chiral metal carbonyl clusters. Notably, while the chiral center is represented by the asymmetric tetrahedral sp^3^ carbon in alpha to the amino acid ligand, the strongest VCD signal is observed in the *ν*_CO_ region. This indicates a transfer of chirality from the organic ligand to the inorganic CO ligands. DFT studies suggest that this transfer is favored by close interactions between the CH_2_ and CH hydrogens of coordinated cysteine and the CO shell of the cluster. In this way, a typical achiral ligand such as CO, may acquire a chiral arrangement around the cluster, becoming active in the VCD spectrum. This envisages the possibility of further extending the application of VCD spectroscopy to chiral carbonyl complexes and clusters. Moreover, the ligand induced symmetry breaking synthetic approach herein presented could be further investigated in order to create more selective catalysts for obtaining organic species.

## Supporting Information

The authors have cited additional references within the Supporting Information.^[^ ^59^, ^60^, ^61^, ^62^, ^63^, ^64^, ^65^, ^66^, ^67^, ^68^ ^]^

## Conflict of Interests

The authors declare no conflict of interest.

## Supporting information



Supporting Information

Supporting Information

Supporting Information

## Data Availability

The data that support the findings of this study are available in the Supporting Information of this article.
