# Supplementary material for: Single‐Step Insertion of Organic Sulfur Into a Fe6C Carbide Carbonyl Cluster, Including the Natural Amino Acid L‐Cysteine: Vibrational Circular Dichroism and Chirality Transfer
Source: Angew Chem Int Ed Engl. 2025 Aug 1;64(38):e202513702. doi: 10.1002/anie.202513702 (PMC12435439; doi:10.1002/anie.202513702)
Supplement: Supplementary file 1 — Supporting Information [file ANIE-64-e202513702-s002.docx]

**Supporting Information for**

**Single-Step Insertion of Organic Sulfur into a Fe_6_C Carbide Carbonyl Cluster, Including the Natural Amino Acid *L*-Cysteine: Vibrational Circular Dichroism and Chirality Transfer**

Francesca Forti, Andrea Pellegrini, Cristiana Cesari, Cristina Femoni, Maria Carmela Iapalucci, Michele Mancinelli and Stefano Zacchini*

Dipartimento di Chimica Industriale "Toso Montanari", Università di Bologna, Via P. Gobetti 85 - 40129 Bologna. Italy. E-mail: [*stefano.zacchini@unibo.it*](mailto:stefano.zacchini@unibo.it)

*Page/s*

Experimental S2-S7

IR spectra S8-S12

NMR spectra S13-S16

Supplementary computational figures and tables S17-S19

Supplementary VCD spectra S20-S21

Supplementary SC-XRD figures and tables S22-S30

References S31

**Experimental**

**General procedures**

All reactions and sample manipulations were carried out under an inert nitrogen atmosphere using standard Schlenk techniques and rigorously dried solvents. All reagents were commercial available with the highest purity and used without further purification, with the exception of compound **1**, which has been synthesized according to the literature.^1^ Analyses of C, H and N were obtained with a Thermo Quest Flash EA 1112NC instrument. IR spectra were recorded on a Perkin Elmer Spectrum One interferometer in CaF_2_ cells. ^1^H and ^13^C{^1^H} NMR measurements were acquired on Varian Inova 600 MHz and Bruker Ascend Avance Neo 600 MHz spectrometers. Chemical shifts for both ¹H and ¹³C{¹H} NMR were referenced to the residual protonated fraction of the solvent. Molecular structures were visualized and illustrated using Mercury software.^2^

**Synthesis of [NEt_4_]_3_[Fe_6_C(CO)_14_(SCH_3_)] (2)**

Dimethyl disulfide (108 μL, 1.21 mmol) was added dropwise in small portions, over a period of 1 h, to a solution of **1** (0.390 g, 0.303 mmol) in CH_3_CN (25 mL). The resulting mixture was stirred at refluxing temperature for 30 min. Then, the solvent was removed under reduced pressure and the residue washed with water (2×20 mL), toluene (2×10 mL), THF (3×10 mL) and extracted, first, with acetone (20 mL) and, then, with CH_3_CN (20 mL). The acetone solution contained a mixture of **2**, **6** and **7**, as evidenced by IR analysis. Purer **2** was present in the CH_3_CN solution. Slow diffusion of n-hexane (2 mL) and di-isopropyl-ether (40 mL) on the CH_3_CN solution afforded crystals of **2** suitable for SC-XRD analyses (0.196 g, yield 55% based on Fe).

**[NEt_4_]_3_[Fe_6_C(CO)_14_(SCH_3_)] (2).** C_40_H_63_Fe_6_N_3_O_14_S (1177.09): calcd. (%): C 40.82, H 5.39, N 3.57; found: C 41.04, H 5.12, N 3.33. FT-IR (CH_3_CN, 298 K) ν_CO_: 1907(s), 1734(w) cm^-1^; FT-IR (nujol mull, 298 K) ν_CO_: 1893(s), 1708(w) cm^-1^. ^1^H NMR (CD_3_CN, 298 K) δ_H_: 3.19 (br, CH_2_, cation), 1.22 (br, CH_3_, cation) ppm. ^13^C{^1^H} NMR (CD_3_CN, 298 K) δ_C_: 237.9 (CO)*, 53.3 (CH_2_, cation), 22.3 (S-CH_3_) 8.0 (CH_3_, cation). *Other carbonyl species present: **1** (δ_C_ = 245.4 ppm), and **6** (δ_C_ = 228.9 ppm).

**Synthesis of [NEt_4_]_3_[Fe_6_C(CO)_14_(SPh)] (3)**

A CH_3_CN solution (2 mL) of phenyl disulfide (30 mg, 0.136 mmol) was added dropwise in small portions, over a period of 1 h, to a solution of **1** (0.350 g, 0.272 mmol) in CH_3_CN (25 mL). The resulting mixture was stirred at room temperature for 1 hour. Then, the solvent was removed under reduced pressure and the residue washed with water (2×20 mL), toluene (2×10 mL), THF (4×10 mL) and extracted, first, with acetone (20 mL) and, then, with CH_3_CN (20 mL). The acetone solution contained a mixture of **3**, **6** and **7**, as evidenced by IR analysis. Purer **3** was present in the CH_3_CN solution. Slow diffusion of n-hexane (2 mL) and di-isopropyl-ether (40 mL) on the CH_3_CN solution afforded crystals of **3** suitable for SC-XRD analyses (0.202 g, yield 60% based on Fe).

**NOTE:** The same compound can be obtained using 1 eq. PhSH added directly as a liquid, at room temperature, instead of PhSSPh.

**[NEt_4_]_3_[Fe_6_C(CO)_14_(SPh)] (3).** C_45_H_65_Fe_6_N_3_O_14_S (1239.16): calcd. (%): C 43.61, H 5.29, N 3.39; found: C 43.39, H 5.40, N 3.08. FT-IR (CH_3_CN, 298 K) ν_CO_: 1914(s), 1733(w) cm^-1^; FT-IR (nujol mull, 298 K) ν_CO_: 1898(s), 1731(w) cm^-1^. ^1^H NMR (CD_3_CN, 298 K) δ_H_: 6.50-7.16 (m, SPh), 3.17 (br, CH_2_, cation), 1.23 (br, CH_3_, cation) ppm. ^13^C{^1^H} NMR (CD_3_CN, 298 K) δ_C_: 237.5 (CO)*, 134.3, 131.2, 127.5, 126.0 (CH, SPh), 54.7 (CH_2_, cation), 9.4 (CH_3_, cation). * Other carbonyl species present: **1** (δ_C_ = 245.4 ppm), **6** (δ_C_ = 228.9 ppm).

**4.4 Synthesis of [NEt_4_]_3_[Fe_6_C(CO)_14_(S-*p*-Tol)] (4)**

A CH_3_CN solution (4 mL) of p-thiocresol (33 mg, 0.260 mmol) was added dropwise in small portions, over a period of 1 h, to a solution of **1** (0.320 g, 0.249 mmol) in CH_3_CN (25 mL). The resulting mixture was stirred at room temperature for 1 hour. Then, the solvent was removed under reduced pressure, and the residue washed with water (3×20 mL), toluene (2×10 mL), THF (3×10 mL) and extracted, first, with acetone (20 mL) and, then, with CH_3_CN (20 mL). The acetone solution contained a mixture of **4**, **6** and **7**, as evidenced by IR analysis. Purer **4** was present in the CH_3_CN solution. Slow diffusion of n-hexane (2 mL) and di-isopropyl-ether (40 mL) on the CH_3_CN solution afforded crystals of **4** suitable for SC-XRD analyses (0.156 g, yield 50% based on Fe).

**NOTE:** A similar reaction was observed by adding 2.5 eq. of p-thiocresol directly as a solid, and heating the solution at refluxing temperature for 1 h. Then, the work-up proceeded as described above.

**[NEt_4_]_3_[Fe_6_C(CO)_14_(S-*p*-Tol)] (4).** C_46_H_67_Fe_6_N_3_O_14_S (1253.18): calcd. (%): C 44.09, H 5.39, N 3.35; found: C 44.22, H 5.11, N 3.07. FT-IR (CH_3_CN, 298 K) ν_CO_: 1914(s), 1718(w) cm^-1^. ^1^H NMR (CD_3_CN, 298 K) δ_H_: 6.59-7.05 (m, p-S*C_6_H_4_*Me), 3.17 (br, CH_2_, cation), 2.16 (s, p-SC_6_H_4_*Me*), 1.24 (br, CH_3_, cation) ppm. ^13^C{^1^H} NMR (CD_3_CN, 298 K) δ_C_: 236.7 (CO)*, 134.9, 133.3, 130.3, 127.4 (CH, p-S*C_6_*H_4_Me), 55.3 (CH_2_, cation), 20.9 (p-SC_6_H_4_*Me*), 10.0 (CH_3_, cation). * Other carbonyl species present: **1** (δ_C_ = 244.6 ppm), **6** (δ_C_ = 228.0 ppm), and **7** (δ_C_ = 226.3, 222.4, 220.2 ppm).

**Synthesis of [NEt_4_]_3_[Fe_6_C(CO)_14_(*L*-Cys)] (*L*-5) and [NEt_4_]_3_[Fe_6_C(CO)_14_(*D*-Cys)] (*D*-5)**

*L*- or *D*-Cysteine (37.3 mg, 0.309 mmol) solubilized in the minimum amount of water, was added drop-wise in small portions, over a period of 2 h, to a solution of **1** (0.398 g, 0.309 mmol) in CH_3_CN (25 mL). The resulting mixture was stirred at room temperature for 1 hour. Then, the solvent was removed under reduced pressure and the residue washed with water (2×10 mL), toluene (2×10 mL), THF (3×10 mL) and extracted, first, with acetone (20 mL) and, then, with CH_3_CN (20 mL). The acetone solution contained a mixture of **5**, **6** and **7**, as evidenced by IR analysis. The CH_3_CN solution contained a purer solution of product **5**, which has been characterized by IR and VCD spectroscopy (0.153 g, yield 40% based on Fe).

**[NEt_4_]_3_[Fe_6_C(CO)_14_(*L*- or *D*-Cys)] (5).** C_45_H_66_Fe_6_N_4_O_16_S (1286.16): calcd. (%): C 42.02, H 5.17, N 4.36; found: C 41.84, H 5.39, N 4.05. FT-IR (CH_3_CN, 298 K) ν_CO_: 1917(s), 1720(w) cm^-1^. ^1^H NMR (CD_3_CN, 298 K) δ_H_: 3.16 (br, CH_2_, cation), 1.20 (br, CH_3_, cation) ppm (the resonances of cysteine are hidden by the broad resonances of cation, solvent and water). ^13^C{^1^H} NMR (CD_3_CN, 298 K) δ_C_: 238.5 (CO)*, 55.4 (CH_2_, cation), 9.5 (CH_3_, cation). *Other carbonyl species present: **1** (δ_C_ = 245.4 ppm), **6** (δ_C_ = 228.8 ppm), and **7** (δ_C_ = 227.1, 223.2, 221.0 ppm). The resonances of cysteine are too weak to be detected.

**VCD studies of [NEt_4_]_3_[Fe_6_C(CO)_14_(*L*-Cys)] (*L*-5) and [NEt_4_]_3_[Fe_6_C(CO)_14_(*D*-Cys)] (*D*-5)**

*Sample preparation*

*L*-cysteine or *D*-cysteine (4.60 mg, 0.0388 mmol) was suspended in D_2_O (0.4 mL), and put in an ultrasound bath for 15 min up to complete dissolution. Then, the cysteine solution was added drop-wise, over a period of 1 hour, to a solution of **1** (0.125 g, 0.0970 mmol) in CH_3_CN (18 mL) kept under Argon atmosphere. The resulting mixture was stirred at room temperature for 30 min, and the reaction was monitored by FT-IR spectroscopy. When the IR spectrum of the reaction mixture showed the ν_CO_ band of **5** as the main product of the reaction, a small portion of the solution was transferred under Ar in the BaF_2_ IR cell used for the VCD analysis.

*VCD Measurement*

Vibrational Circular Dichroism (VCD) measurements were performed on a ChiralIR-2X FT-VCD spectrometer (Biotools, Inc.) equipped with single PEM operating at 36 kHz and a resolution of 8 cm^-1^, with parameters optimized for 1600 cm^-1^, interfaced to a computer employing ChiralIR-2X software and Grams AI for data treatment. Every different experimental condition was tested at least twice from independent samples, employing distilled and degassed CH_3_CN as the solvent of the cluster and D_2_O as solvent for the *L*- and *D*-cysteine. Samples were loaded under Ar atmosphere in a BaF_2_ IR cell with two windows separated by a 0.1 mm Teflon spacer; the approximate concentration of all cluster samples was around 3.5 mM, affording a IR relative absorbance between 0.3 and 0.6 units. The final spectra were averaged from at least 3 blocks, each composed of 3500 interferometric scans accumulated for 30 min or more. Baseline corrections using the spectra of the relevant solvent and blank tests, obtained under the same conditions, were performed. The final VCD spectrum of each enantiomer was obtained by subtracting the spectrum of the opposite enantiomer and halving the result. This approach effectively removes artifacts arising from the solvent, cell positioning and geometry, and for baseline irregularities.

**DFT Studies**

*Clusters’ optimization and qualitative analysis*

All geometries here reported have been optimized using the composite method PBEh-3c ^3^ using a triple zeta basis set (def2-TZVPP) ^4^ using the C-PCM ^5^ as implicit solvation model, when solution is considered. The level of theory has been chosen based on previous calculation on similar structures,^6^ and confirmed by comparing the optimized structures of **2-4** with those determined by SC-XRD based on RMSD values (Table S1). To remove the complexity of the ionic pair, the three [NEt_4_]^+^ cations have been removed. Due to the highly charged system, a big and diffused basis set has been used (def2-QZVPPD) and compared to a smaller basis set (def2-TZVPP). From the accordance and the small variation of these values, the work proceeded employing the more manageable def2-TZVPP basis set.

For the calculation of the critical points the software used was MultiWFN,^7^ while for Non-Covalent Interaction index surfaces the NCIPlot ^8^ software (version 4.2) was employed.

*VCD simulations*

DFT calculations to obtain simulated VCD spectra of compound ***L*-5** were carried out employing a molecular dynamic calculation at GFN1-xTB on the [Fe_6_C(CO)_14_(*L*-Cys)]^3–^ geometry, including CH_3_CN as implicit solvation with the ALPB model.^6^ The geometries above 4 kcal/mol or the duplicated geometries have been sorted out. For all the remaining 22 conformers, the vibrational graphs – both IR and VCD - have been computed, weighted on the Boltzmann population and convoluted using a Lorentzian line shape for each transition have been used, using 20 cm^-1^ as full width half maximum. From this calculation 8 snapshots have been considered and optimized with ORCA 5.0.4 to PBEh-3cdef2-TZVP, including CH_3_CN as implicit solvation thanks to the C-PCM model. Using Gaussian16 Revision A.03, the 8 snapshots where furthermore refined at wb97xddef2-TZVP level maintaining CH_3_CN as implicit solvent with C-PCM model.

To simulate VCD and IR spectra of free *L*-cysteine, its zwitterionic form has been computed after a conformational search, conducted at the GFN2-xTB ^6^ level using GOAT algorithm in ORCA (version 6.0).^9^ The conformational space has been reduced using the Principal Component Analysis on the eigenvalues of the distance matrix and clustering the conformers in 30 groups.

**X-ray Crystallographic Study**

Crystal data and collection details for the new clusters **2-4**, and the side products [NEt_4_]_5_[Fe_6_C(CO)_15_][Cl], and [NEt_4_]_3_[Fe_4_C(CO)_12_][Cl]**·**H_2_O are reported in Table S5. [NEt_4_]_5_[Fe_6_C(CO)_15_][Cl], [NEt_4_]_3_[Fe_4_C(CO)_12_][Cl]**·**H_2_O have been isolated as side products during the work-up of some of the reactions which afforded **2-5**, and contain the cluster anions **1** and **8**, respectively, already known in the literature.

The diffraction experiments were carried out on a Bruker APEX II diffractometer equipped with a PHOTON2 detector using Mo–Kα radiation. Data were corrected for Lorentz polarization and absorption effects (empirical absorption correction SADABS).^10^ Structures were solved by direct methods and refined by full-matrix least-squares based on all data using *F*^2^.^11^ Hydrogen atoms were fixed at calculated positions and refined by a riding model, unless otherwise stated.

**Figure S1.** IR spectrum in the ν_CO_ region of [NEt_4_]_2_[Fe_6_C(CO)_16_] (**6**) recorded in CH_3_CN.

**Figure S2.** IR spectrum in the ν_CO_ region of [NEt_4_]_4_[Fe_6_C(CO)_15_] (**1**) recorded in CH_3_CN.

**Figure S3.** IR spectrum in the ν_CO_ region of [NEt_4_]_2_[Fe_5_C(CO)_14_] (**7**) recorded in THF.

**Figure S4.** IR spectrum in the ν_CO_ region of [NEt_4_]_2_[Fe_4_C(CO)_12_] (**8**) recorded in THF.

**Figure S5.** IR spectrum in the ν_CO_ region of [NEt_4_]_3_[Fe_6_C(CO)_14_(SMe)] (**2**) recorded in CH_3_CN.

**Figure S6.** IR spectrum of [NEt_4_]_3_[Fe_6_C(CO)_14_(SMe)] (**2**) in nujol mull.

**Figure S7.** IR spectrum in the ν_CO_ region of [NEt_4_]_3_[Fe_6_C(CO)_14_(SPh)] (**3**) recorded in CH_3_CN.

**Figure S8.** IR spectrum of [NEt_4_]_3_[Fe_6_C(CO)_14_(SPh)] (**3**) in nujol mull.

**Figure S9.** IR spectrum in the ν_CO_ region of [NEt_4_]_3_[Fe_6_C(CO)_14_(p-SC_6_H_4_Me)] (**4**) recorded in CH_3_CN.

**Figure S10.** IR spectrum in the ν_CO_ region of [NEt_4_]_3_[Fe_6_C(CO)_14_(*L*-Cys)] (***L*-5**) recorded in CH_3_CN.

**Figure S11.** ^1^H NMR spectrum of [NEt_4_]_3_[Fe_6_C(CO)_14_(SMe)] (**2**) recorded in CD_3_CN at 298 K.

**Figure S12.** ^13^C{^1^H} NMR spectrum of [NEt_4_]_3_[Fe_6_C(CO)_14_(SMe)] (**2**) recorded in CD_3_CN at 298 K.

^*^ [NEt_4_]_4_[Fe_6_C(CO)_15_] (**1**); ^✝^ [NEt_4_]_2_[Fe_6_C(CO)_16_] (**6**)

**Figure S13.** ^1^H NMR spectrum of [NEt_4_]_3_[Fe_6_C(CO)_14_(SPh)] (**3**) recorded in CD_3_CN at 298 K.

**Figure S14.** ^13^C{^1^H} NMR spectrum of [NEt_4_]_3_[Fe_6_C(CO)_14_(SPh)] (**3**) recorded in CD_3_CN at 298 K.

^*^ [NEt_4_]_4_[Fe_6_C(CO)_15_] (**1**); ^✝^ [NEt_4_]_2_[Fe_6_C(CO)_16_] (**6**)

**Figure S15.** ^1^H NMR spectrum of [NEt_4_]_3_[Fe_6_C(CO)_14_(p-SC_6_H_4_Me)] (**4**) recorded in CD_3_CN at 298 K.

**Figure S16.** ^13^C{^1^H} NMR spectrum of [NEt_4_]_3_[Fe_6_C(CO)_14_(p-SC_6_H_4_Me)] (**4**) recorded in CD_3_CN at 298 K.

^*^ [NEt_4_]_4_[Fe_6_C(CO)_15_] (**1**); ^✝^ [NEt_4_]_2_[Fe_6_C(CO)_16_] (**6**); ^#^ [NEt_4_]_2_[Fe_5_C(CO)_14_] (**7**)

**Figure S17.** ^1^H NMR spectrum of [NEt_4_]_3_[Fe_6_C(CO)_14_(*L*-Cys)] (***L*-5**) recorded in CD_3_CN at 298 K.

**Figure S18.** ^13^C{^1^H} NMR spectrum of [NEt_4_]_3_[Fe_6_C(CO)_14_(*L*-Cys)] (***L*-5**) recorded in CD_3_CN at 298 K.

^*^ [NEt_4_]_4_[Fe_6_C(CO)_15_] (**1**); ^✝^ [NEt_4_]_2_[Fe_6_C(CO)_16_] (**6**); ^#^ [NEt_4_]_2_[Fe_5_C(CO)_14_] (**7**)


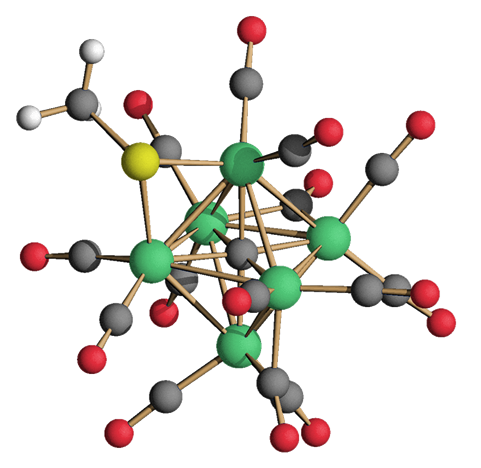


**Figure S19**. DFT-optimized structure of [Fe_6_C(CO)_14_(SMe)]^3–^ (**2**) (green Fe; red O; grey C; yellow S; white H).


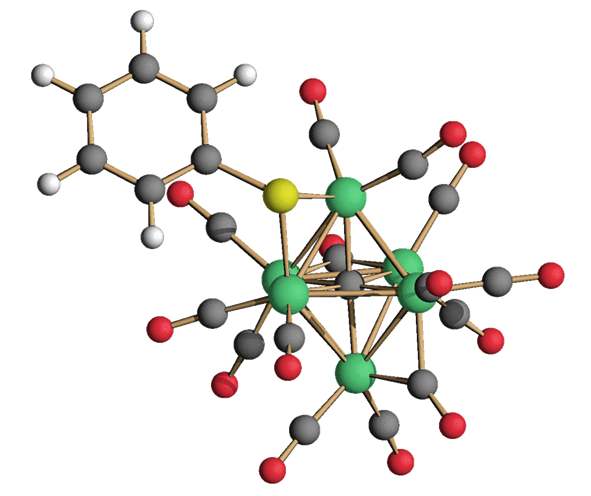


**Figure S20**. DFT-optimized structure of [Fe_6_C(CO)_14_(SPh)]^3–^ (**3**) (green Fe; red O; grey C; yellow S; white H).

**
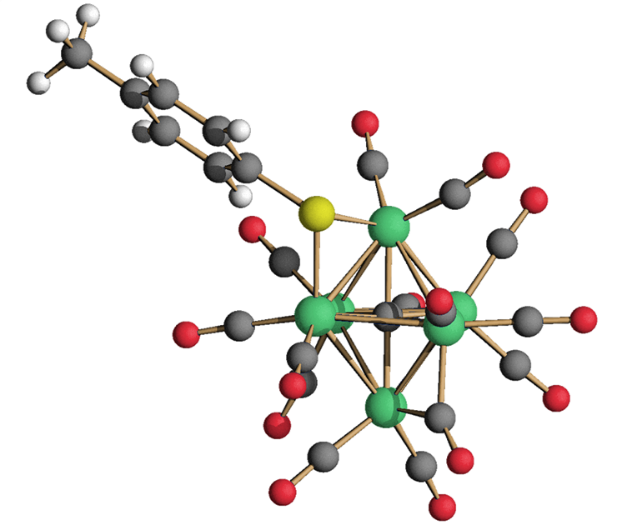
**

**Figure S21**. DFT-optimized structure of [Fe_6_C(CO)_14_(p-SC_6_H_4_Me)]^3–^ (**4**) (green Fe; red O; grey C; yellow S; white H).


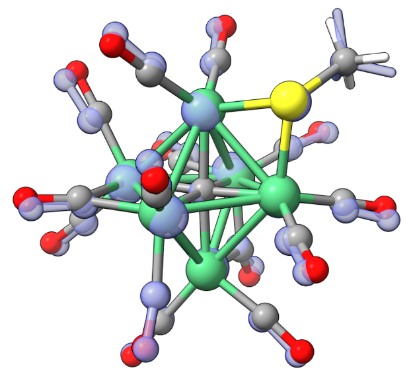


**Figure S22.** Best superimposition of the SC-XRD molecular structure of [Fe_6_C(CO)_14_(SMe)]^3–^ (**2**) (green Fe; red O; grey C; yellow S; white H) and DFT-optimized structure (clear light blue).


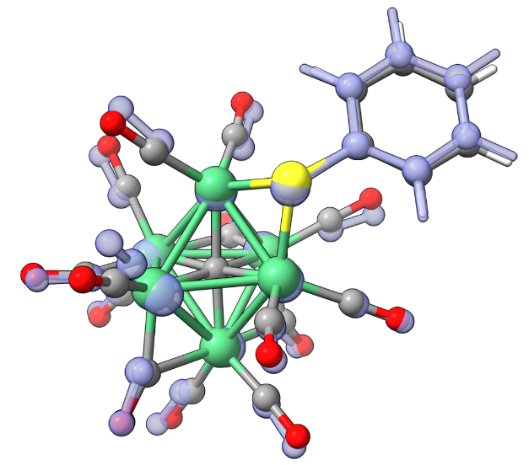


**Figure S23.** Best superimposition of the SC-XRD molecular structure of [Fe_6_C(CO)_14_(SPh)]^3–^ (**3**) (green Fe; red O; grey C; yellow S; white H) and DFT-optimized structure (clear light blue).


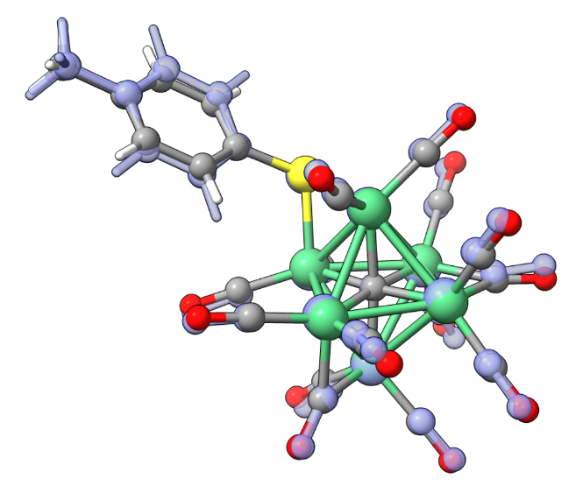


**Figure S24.** Best superimposition of the SC-XRD molecular structure of [Fe_6_C(CO)_14_(p-SC_6_H_4_Me)]^3–^ (**4**) (green Fe; red O; grey C; yellow S; white H) and DFT-optimized structure (clear light blue).

**Table S1**. Atomic RMSD values; comparison between the SC-XRD structures and the computed geometry at ωB97x-D4 with different basis set.

| **RMSD (Å)** | **def2-TZVPP** | **def2-QZVPPD** |
| --- | --- | --- |
| [Fe_6_C(CO)_14_(SMe)]^3–^ | 0.25 | 0.28 |
| [Fe_6_C(CO)_14_(SPh)]^3–^ | 0.32 | 0.44 |
| [Fe_6_C(CO)_14_(S-*p*-Tol)]^3–^ | 0.38 | 0.40 |

**Table S2.** Gibbs Free energy and Boltzmann population of the 8 conformers of the snapshot geometries considered for the VCD simulation of [Fe_6_C(CO)_14_(*L*-Cysteine)]^3–^ (***L*-5**), calculated at standard condition (1 M, 1 bar and 298 K).

| **Conformer** | **ΔG [kcal/mol]** | **Population (%)** |
| --- | --- | --- |
| 1 | 0.00 | 12.86 |
| 2 | 0.02 | 12.41 |
| 3 | 0.02 | 12.45 |
| 4 | 0.02 | 12.53 |
| 5 | 0.02 | 12.38 |
| 6 | 0.02 | 12.41 |
| 7 | 0.03 | 12.30 |
| 8 | 0.01 | 12.66 |


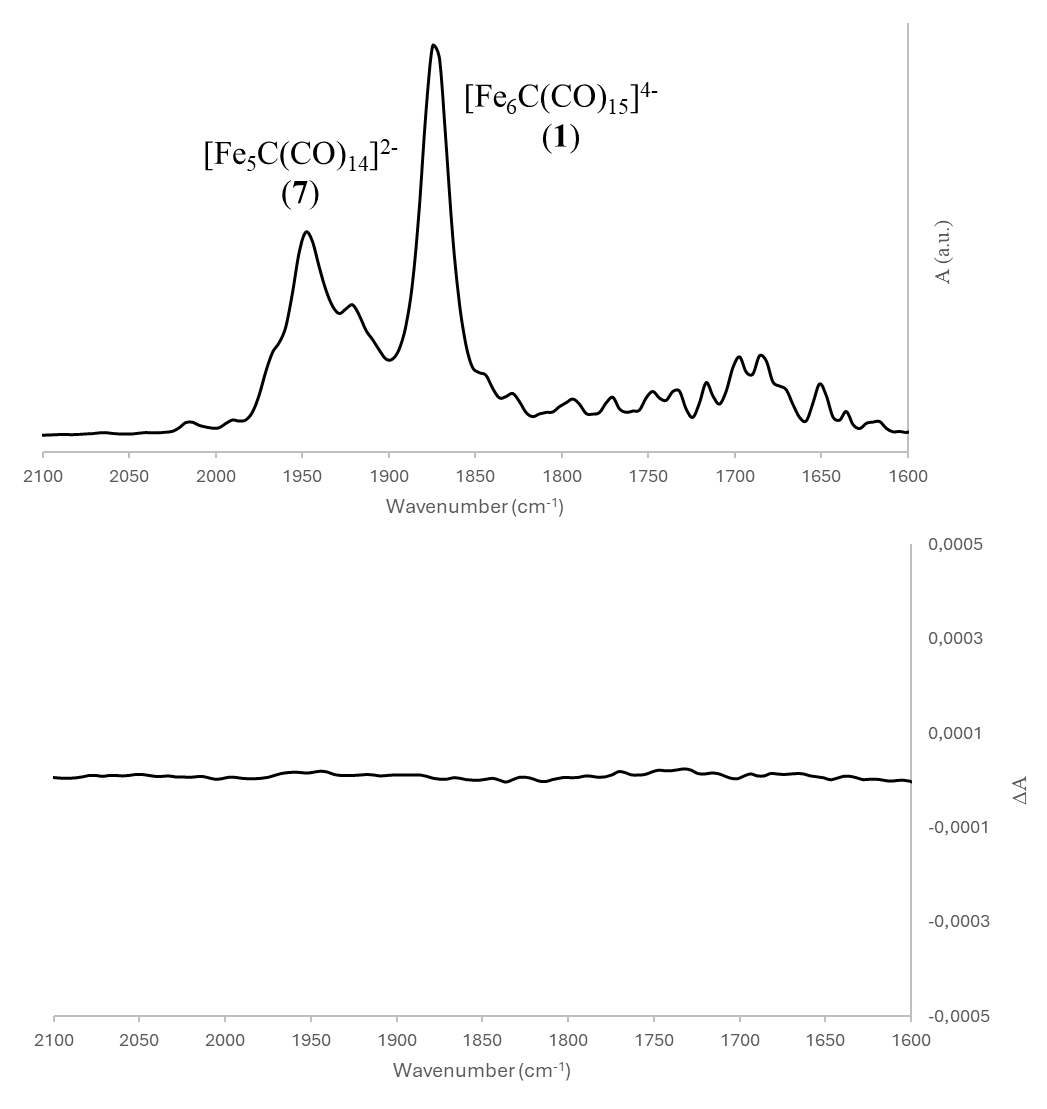


**Figure S25.** Experimental IR (top) and VCD (bottom) spectra of [NEt_4_]_2_[Fe_6_C(CO)_15_] (**1**) in CH_3_CN.


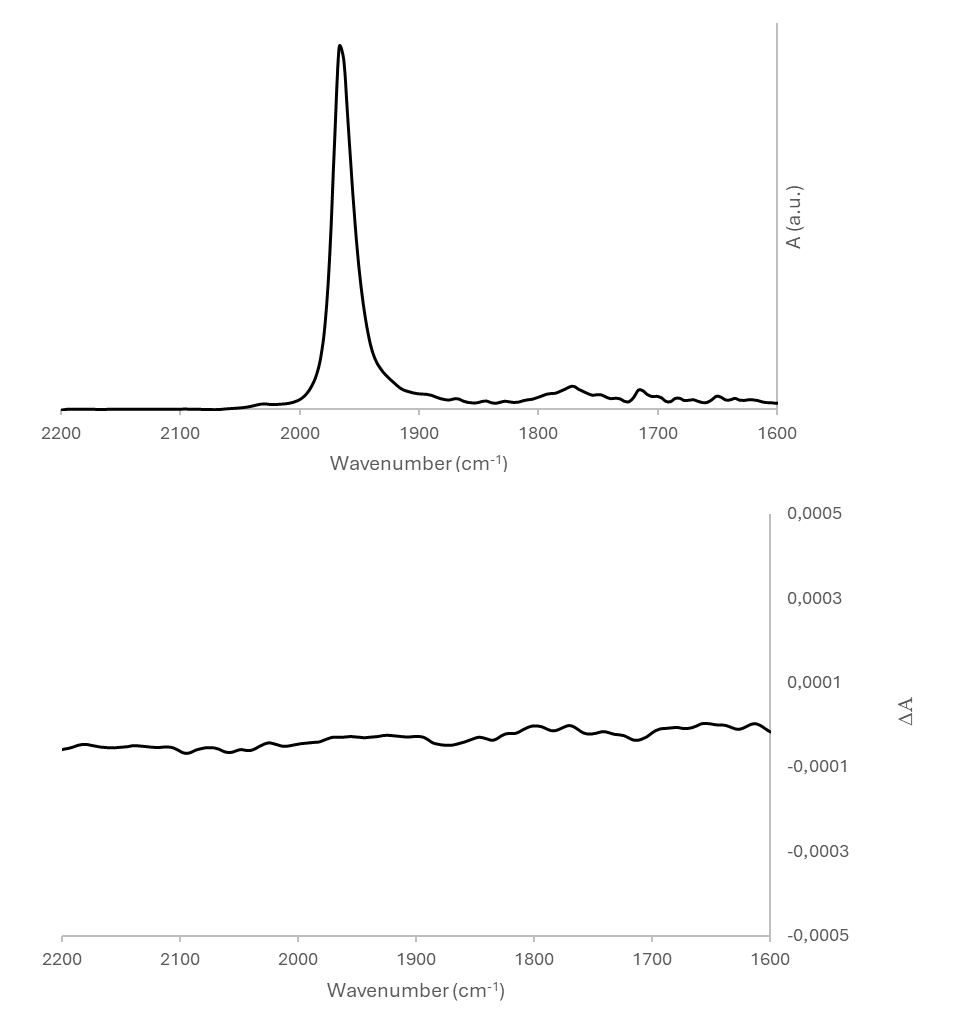


**Figure S26.** Experimental IR (top) and VCD (bottom) spectra of [NEt_4_]_2_[Fe_6_C(CO)_16_] (**6**) in CH_3_CN.


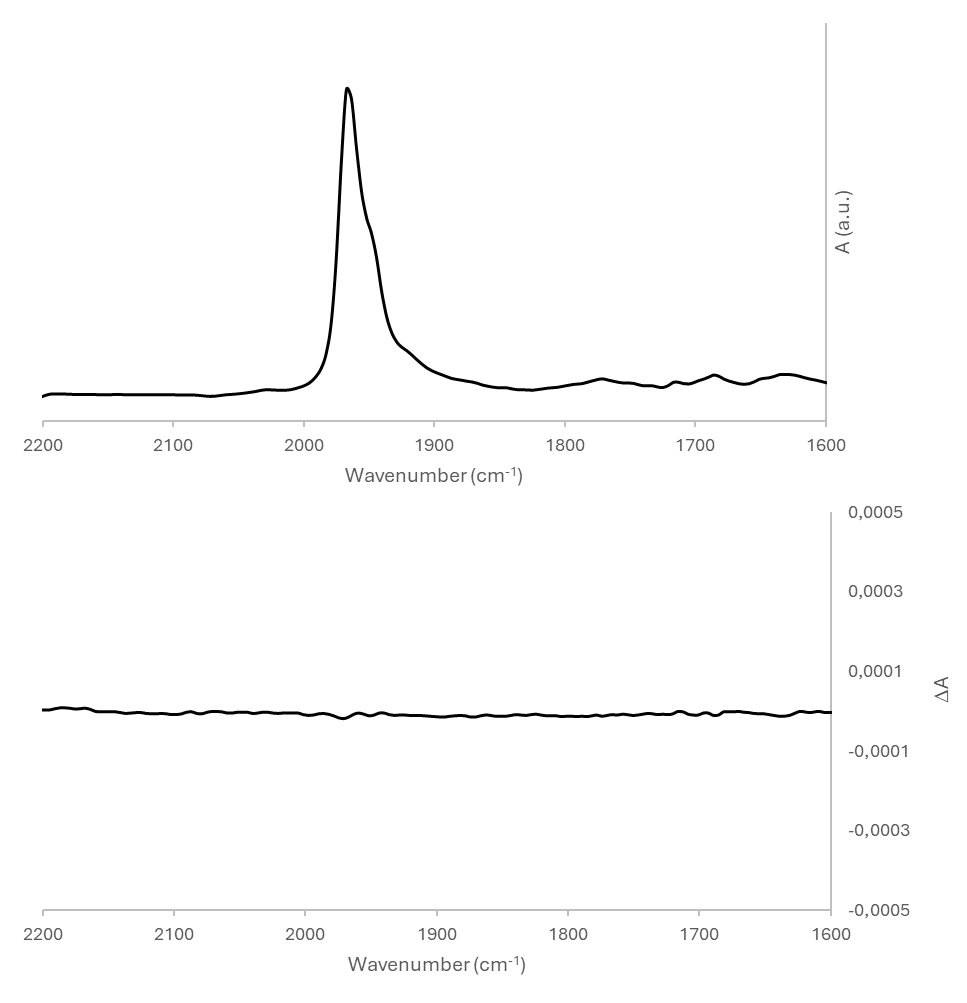


**Figure S27.** Experimental IR (top) and VCD (bottom) spectra of [NEt_4_]_2_[Fe_5_C(CO)_14_] (**7**) in CH_3_CN.


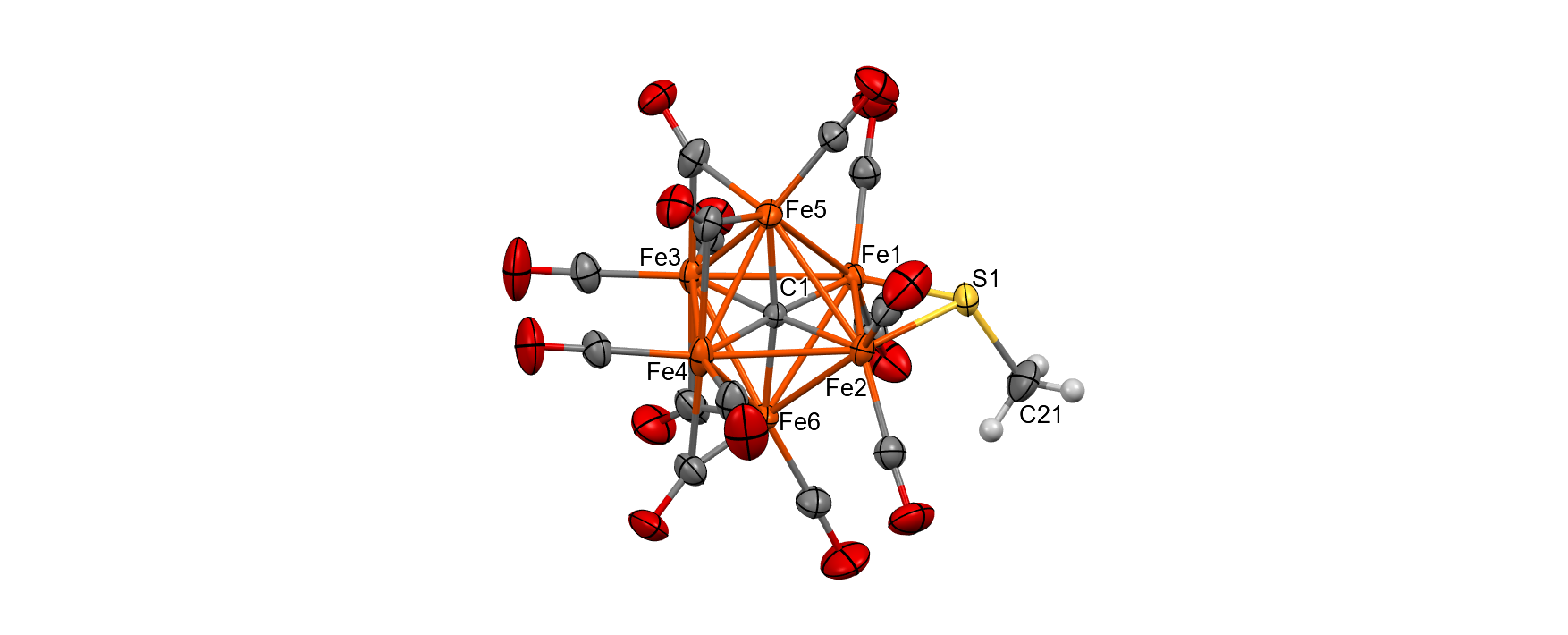


**Figure S28.** Molecular structure of [Fe_6_C(CO)_14_(SMe)]^3–^ as found in [NEt_4_]_3_[Fe_6_C(CO)_14_(SMe)] (**2**) (orange, Fe; yellow, S; red, O; grey, C; white, H). Thermal ellipsoids are at the 30% probability level. SC-XRD data collected at 293 K.


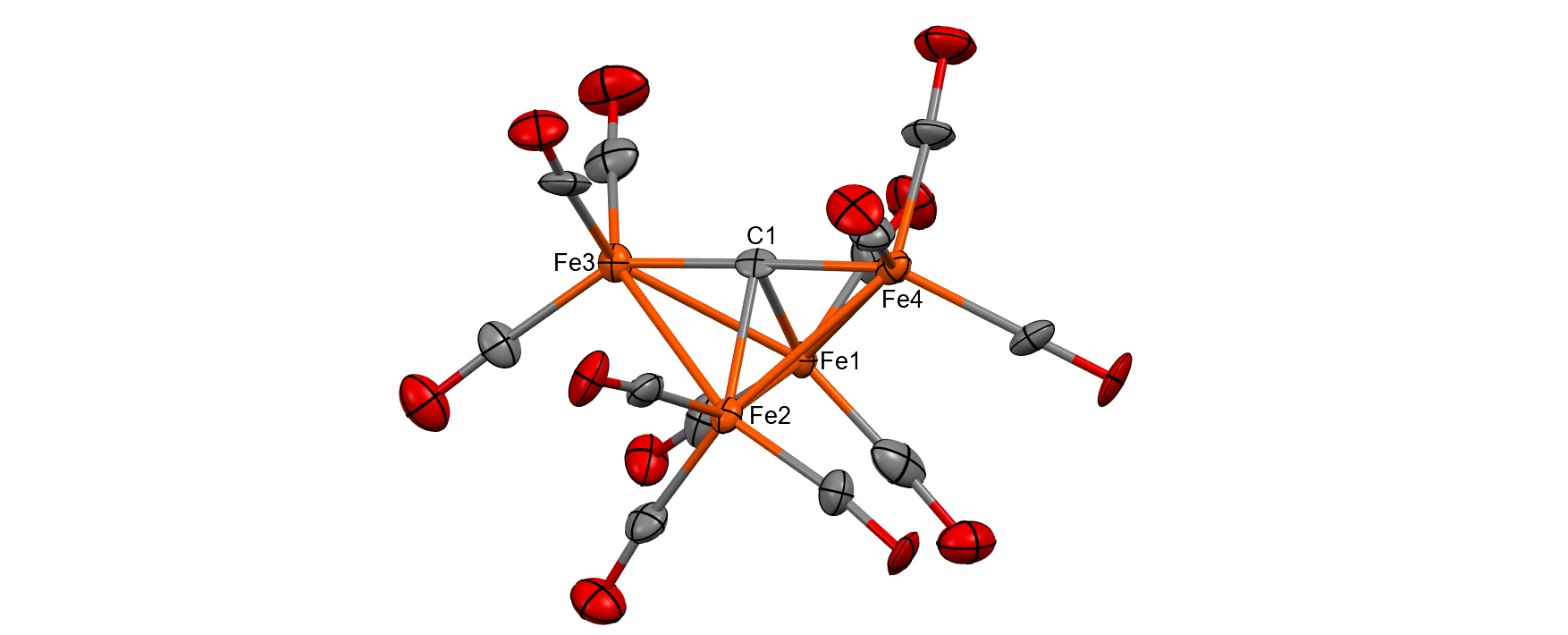


**Figure S29.** Molecular structure of [Fe_4_C(CO)_12_]^2–^ (**8**) as found in [NEt_4_]_3_[Fe_4_C(CO)_12_][Cl]**·**H_2_O (orange, Fe; red, O; grey, C; white, H). Thermal ellipsoids are at the 30% probability level. SC-XRD data collected at 100 K.


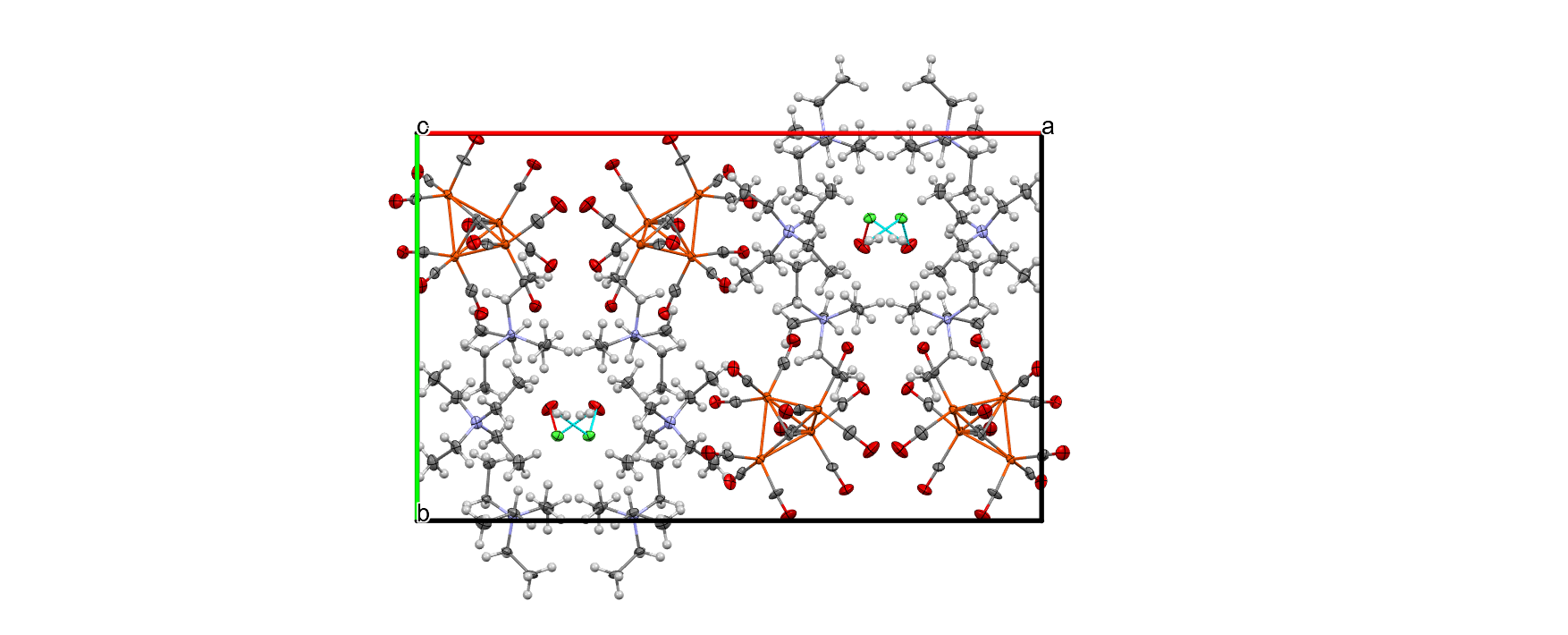


**Figure S30.** Crystal packing of [NEt_4_]_3_[Fe_4_C(CO)_12_][Cl]**·**H_2_O viewed along the crystallographic *c*-axis (orange, Fe; green, Cl; red, O; grey, C; white, H). Thermal ellipsoids are at the 30% probability level. SC-XRD data collected at 100 K. H-bonds are represented as dotted blue lines.


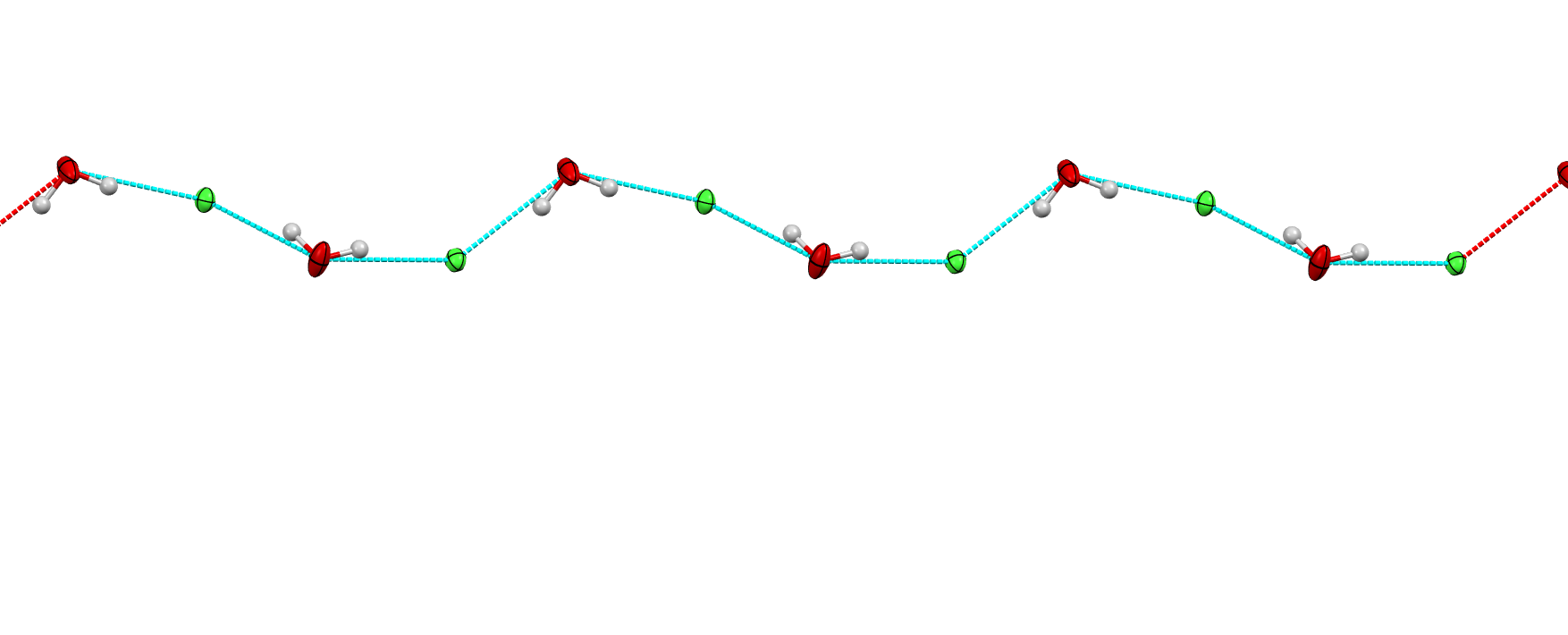


**Figure S31.** Hydrogen bond network involving the H_2_O molecules and Cl^–^ anions as found within the crystal structure of [NEt_4_]_3_[Fe_4_C(CO)_12_][Cl]**·**H_2_O viewed along the crystallographic *c*-axis (green, Cl; red, O; white, H). Thermal ellipsoids are at the 30% probability level. SC-XRD data collected at 100 K. H-bonds are represented as dotted blue lines.


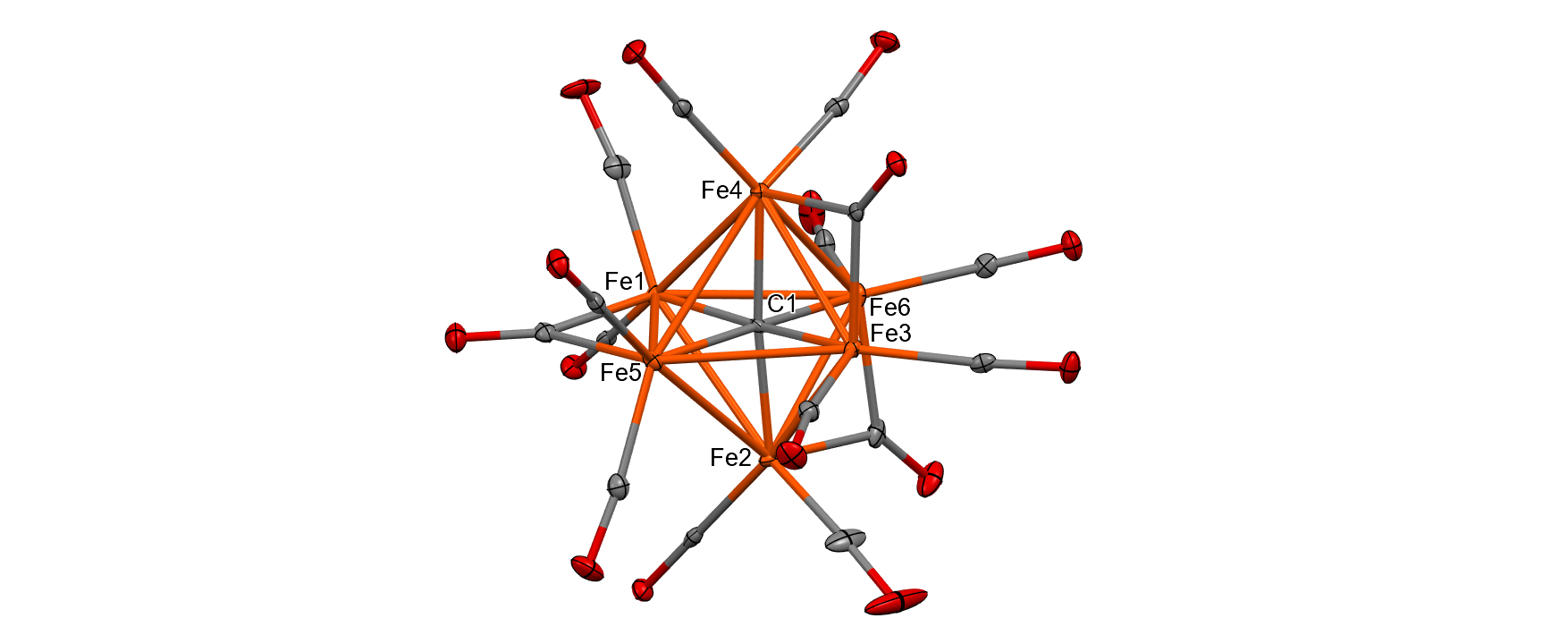


**Figure S32.** Molecular structure of [Fe_6_C(CO)_15_]^4–^ (**1**) as found in [NEt_4_]_5_[Fe_6_C(CO)_15_][Cl] (orange, Fe; red, O; grey, C; white, H). Thermal ellipsoids are at the 30% probability level. SC-XRD data collected at 100 K.

**Table S3.** Bond distances (Å) and angles (°) of **2-4**. All SC-XRD data have been collected at 100 K, unless otherwise stated.

|  | **2** | **3** | **4** | **2 (293 K)** |
| --- | --- | --- | --- | --- |
| Fe(1)-Fe(2) | 2.5621(6) | 2.551(2) | 2.568(2) | 2.5586(11) |
| Fe(1)-Fe(3) | 2.6648(6) | 2.646(3) | 2.678(2) | 2.6473(11) |
| Fe(1)-Fe(5) | 2.7145(6) | 2.736(2) | 2.7055(18) | 2.6894(11) |
| Fe(1)-Fe(6) | 2.7346(7) | 2.674(3) | 2.7071(17) | 2.7440(11) |
| Fe(2)-Fe(4) | 2.6476(6) | 2.645(3) | 2.647(2) | 2.6646(12) |
| Fe(2)-Fe(5) | 2.6911(7) | 2.730(3) | 2.6627(19) | 2.7122(11) |
| Fe(2)-Fe(6) | 2.7417(6) | 2.687(3) | 2.7555(17) | 2.7197(12) |
| Fe(3)-Fe(4) | 2.7656(7) | 2.723(3) | 2.739(2) | 2.7538(12) |
| Fe(3)-Fe(5) | 2.6410(7) | 2.557(3) | 2.5608(17) | 2.5871(12) |
| Fe(3)-Fe(6) | 2.5574(7) | 2.610(4) | 2.679(2) | 2.6159(12) |
| Fe(4)-Fe(5) | 2.5651(7) | 2.586(3) | 2.7087(17) | 2.6210(13) |
| Fe(4)-Fe(6) | 2.6398(7) | 2.609(3) | 2.5553(19) | 2.5628(13) |
| Fe(1)-S(1) | 2.2130(9) | 2.215(3) | 2.2216(19) | 2.1980(18) |
| Fe(2)-S(1) | 2.2016(10) | 2.216(3) | 2.2048(18) | 2.2134(17) |
| Fe(1)-C(1) | 1.881(3) | 1.902(12) | 1.886(5) | 1.871(5) |
| Fe(2)-C(1) | 1.874(3) | 1.846(15) | 1.875(5) | 1.869(5) |
| Fe(3)-C(1) | 1.881(3) | 1.887(15) | 1.878(5) | 1.893(5) |
| Fe(4)-C(1) | 1.890(3) | 1.840(12) | 1.878(5) | 1.881(5) |
| Fe(5)-C(1) | 1.873(3) | 1.865(10) | 1.878(5) | 1.868(5) |
| Fe(6)-C(1) | 1.886(3) | 1.888(10) | 1.889(5) | 1.886(5) |
| Fe-C(O)_terminal_ range | 1.745(4)-1.771(4) | 1.72(2)-1.803(17) | 1.742(6)-1.793(6) | 1.733(6)-1.767(7) |
| Fe-C(O)_terminal_ average | 1.759(13) | 1.76(5) | 1.759(19) | 1.75(2) |
| Fe(5)-C(2) | 1.887(3) | 1.841(12) | 1.762(6) | 1.786(7) |
| Fe(4)-C(2) | 2.034(4) | 2.257(16) | 2.974(6) | 2.422(7) |
| Fe(5)-C(3) | 1.766(4) | 1.816(17) | 1.911(5) | 1.851(7) |
| Fe(3)-C(3) | 2.553(4) | 2.213(15) | 1.976(5) | 2.182(8) |
| Fe(6)-C(4) | 1.910(3) | 1.796(17) | 1.756(6) | 1.800(8) |
| Fe(3)-C(4) | 1.987(3) | 2.451(17) | 2.718(6) | 2.386(9) |
| Fe(6)-C(5) | 1.768(4) | 1.846(18) | 1.929(5) | 1.865(7) |
| Fe(4)-C(5) | 2.524(4) | 2.33(2) | 1.941(5) | 2.121(7) |
| α_CO(2)_ | 0.078(3) | 0.226(12) | 0.688(7) | 0.356(7) |
| α_CO(3)_ | 0.446(4) | 0.219(14) | 0.034(4) | 0.179(6) |
| α_CO(4)_ | 0.040(2) | 0.365(16) | 0.548(6) | 0.326(8) |
| α_CO(5)_ | 0.428(4) | 0.262(16) | 0.006(4) | 0.137(6) |
| Fe(5)-C(2)-O(2) | 144.5(3) | 154.4(15) | 173.4(5) | 159.9(6) |
| Fe(5)-C(3)-O(3) | 166.5(3) | 150.2(12) | 140.8(4) | 149.5(7) |
| Fe(6)-C(4)-O(4) | 142.4(3) | 157.7(14) | 171.3(5) | 158.5(8) |
| Fe(6)-C(5)-O(5) | 164.6(3) | 150.6(19) | 139.3(4) | 148.2(7) |
| Distance of S(1) from the Fe(1)Fe(2)Fe(3)Fe(4) least squares plane | 0.212 | 0.0020 | 0.424 | 0.212 |
| Angle between the Fe(1)Fe(2)S(1) and Fe(1)Fe(2)Fe(5) planes | 121.6 | 129.2 | 113.5 | 121.5 |
| Fe(5)-C(1)-Fe(6) | 172.35(18) | 172.8(7) | 175.1(3) | 172.0(3) |

**Table S4.** Main bond distances (Å) of [NEt_4_]_3_[Fe_6_C(CO)_14_(SMe)] (**2**), [NEt_4_]_3_[Fe_6_C(CO)_14_(SPh)] (**3**), and [NEt_4_]_3_[Fe_6_C(CO)_14_(S-*p*-Tol)] (**4**), compared to [NEt_4_]_5_[Fe_6_C(CO)_15_][Cl], [NMe_3_CH_2_Ph]_4_[Fe_6_C(CO)_15_] (two different polymorphs, both with space group *C*2/*c*), [NEt_4_]_4_[Fe_6_C(CO)_15_]**·**CH_3_CN, [NMe_3_CH_2_Ph]_2_[Fe_6_C(CO)_16_], [NEt_4_]_3_[H_3_O][Fe_6_C(CO)_14_(CO_3_)] (two independent crystals have been collected), [NEt_4_]_2_[Fe_6_C(CO)_15_(PTA)]. All SC-XRD data have been collected at 100 K, unless otherwise stated.

|  | **Fe-Fe** | **Fe-C_carbide_** |
| --- | --- | --- |
| [NEt_4_]_3_[Fe_6_C(CO)_14_(SMe)] | 2.5574(7)-2.7656(7)  Average 2.660(2) | 1.873(3)-1.890(3)  Average 1.881(7) |
| [NEt_4_]_3_[Fe_6_C(CO)_14_(SMe)] (293 K) | 2.5586(11)-2.7538(12)  Average 2.656(4) | 1.868(5)-1.893(5)  Average 1.878(12) |
| [NEt_4_]_3_[Fe_6_C(CO)_14_(SPh)] | 2.551(2)-2.736(2)  Average 2.646(7) | 1.840(12)-1.902(12)  Average 1.87(3) |
| [NEt_4_]_3_[Fe_6_C(CO)_14_(S-*p*-Tol)] | 2.5553(19)-2.7555(17)  Average 2.664(6) | 1.875(5)-1.889(5)  Average 1.881(12) |
| [NEt_4_]_5_[Fe_6_C(CO)_6_][Cl] | 2.5461(5)-2.7339(6)  Average 2.659(2) | 1.867(3)-1.888(3)  Average 1.880(7) |
| [NMe_3_CH_2_Ph]_4_[Fe_6_C(CO)_15_]  *C*2/*c*, polymorph 1 ^a^ | 2.5283(18)-2.7248(18)  Average 2.646(7) | 1.862(7)-1.882(7)  Average 1.87(2) |
| [NMe_3_CH_2_Ph]_4_[Fe_6_C(CO)_15_]  *C*2/*c*, polymorph 2 ^a^ | 2.5509(6)-2.7329(6)  Average 2.659(2) | 1.872(3)-1.890(3)  Average 1.880(7) |
| [NEt_4_]_4_[Fe_6_C(CO)_15_]**·**CH_3_CN ^b^ | 2.5394(7)-2.7914(8)  Average 2.6612(18) | 1.8710(4)-1.889(2)  Average 1.880(3) |
| [NMe_3_CH_2_Ph]_2_[Fe_6_C(CO)_16_] ^a^ | 2.5682(11)-2.7114(10)  Average 2.664(3) | 1.871(5)-1.897(5)  Average 1.884(12) |
| [NEt_4_]_3_[H_3_O][Fe_6_C(CO)_14_(CO_3_)] ^a^  Crystal 1 | 2.5689(4)-2.7915(4)  Average 2.6886(14) | 1.876(2)-1.9443(19)  Average 1.902(5) |
| [NEt_4_]_3_[H_3_O][Fe_6_C(CO)_14_(CO_3_)]  Crystal 2 ^a^ | 2.5674(7)-2.7893(7)  Average 2.687(2) | 1.869(3)-1.941(3)  Average 1.901(7) |
| [NEt_4_]_2_[Fe_6_C(CO)_15_(PTA)] ^a^ | 2.559(5)-2.914(5)  Average 2.67(2) | 1.84(2)-1.93(2)  Average 1.89(7) |

^a^ From ref. 12. ^b^ From ref. 1.

**Table S5.** Crystal data and experimental details for [NEt_4_]_3_[Fe_6_C(CO)_14_(SMe)], [NEt_4_]_3_[Fe_6_C(CO)_14_(SPh)], [NEt_4_]_3_[Fe_6_C(CO)_14_(S-*p*-Tol)], [NEt_4_]_5_[Fe_6_C(CO)_15_][Cl], [NEt_4_]_3_[Fe_4_C(CO)_12_][Cl]**·**H_2_O.

|  | **[NEt_4_]_3_[Fe_6_C(CO)_14_(SMe)]** | **[NEt_4_]_3_[Fe_6_C(CO)_14_(SPh)]** | **[NEt_4_]_3_[Fe_6_C(CO)_14_(S-*p*-Tol)]** |
| --- | --- | --- | --- |
| Formula | C_40_H_63_Fe_6_N_3_O_14_S | C_45_H_65_Fe_6_N_3_O_14_S | C_46_H_67_Fe_6_N_3_O_14_S |
| *Fw* | 1177.09 | 1239.16 | 1253.18 |
| T, K | 100(2) | 100(2) | 100(2) |
| λ, Å | 0.71073 | 0.71073 | 0.71073 |
| Crystal system | Orthorhombic | Tetragonal | Monoclinic |
| Space Group | *Pna*2_1_ | *I*4_1_*cd* | *P*2_1_/*c* |
| a, Å | 13.2324(7) | 27.415(2) | 17.078(15) |
| b, Å | 20.6465(11) | 27.415(2) | 12.009(8) |
| c, Å | 17.8771(9) | 27.1058(19) | 26.037(19) |
| α, ° | 90 | 90 | 90 |
| β, ° | 90 | 90 | 90.64(3) |
| γ, ° | 90 | 90 | 90 |
| Cell Volume, Å^3^ | 4884.1(4) | 20373(3) | 5340(7) |
| Z | 4 | 16 | 4 |
| D_c_, g cm^-3^ | 1.601 | 1.616 | 1.559 |
| μ, mm^-1^ | 1.842 | 1.771 | 1.690 |
| F(000) | 2432 | 10240 | 2592 |
| Crystal size, mm | 0.22×0.16×0.14 | 0.22×0.19×0.10 | 0.21×0.16×0.10 |
| θ limits, ° | 1.828-26.994 | 1.823-25.998 | 1.867–25.999 |
| Index ranges | -16 ≤ h ≤ 16  -26 ≤ k ≤ 26  -22 ≤ l ≤ 22 | -33 ≤ h ≤ 33  -33 ≤ k ≤ 33  -33 ≤ l ≤ 33 | -21 ≤ h ≤ 21  -14 ≤ k ≤ 14  -32 ≤ l ≤ 32 |
| Reflections collected | 70196 | 132504 | 68419 |
| Independent reflections | 10661 [R_int_ = 0.0685] | 9960 [R_int_ = 0.0713] | 10489 [R_int_ = 0.0784] |
| Completeness to θ max | 99.9% | 99.8% | 99.8% |
| Data / restraints / parameters | 10661 / 241 / 627 | 9960 / 684 / 757 | 10489 / 256 / 697 |
| Goodness on fit on F^2^ | 1.043 | 1.153 | 1.073 |
| R_1_ (I > 2σ(I)) | 0.0250 | 0.0816 | 0.0641 |
| wR_2_ (all data) | 0.0633 | 0.1858 | 0.1714 |
| Largest diff. peak and hole, e Å^-3^ | 0.554 / -0.421 | 1.273 / -0.872 | 1.589 / –0.793 |

|  | **[NEt_4_]_5_[Fe_6_C(CO)_15_][Cl]** | **[NEt_4_]_3_[Fe_4_C(CO)_12_][Cl]·H_2_O** | **[NEt_4_]_3_[Fe_6_C(CO)_14_(SMe)] (293 K)** |
| --- | --- | --- | --- |
| Formula | C_56_H_100_ClFe_6_N_5_O_15_ | C_37_H_62_ClFe_4_N_3_O_13_ | C_40_H_63_Fe_6_N_3_O_14_S |
| *Fw* | 1453.95 | 1015.74 | 1177.09 |
| T, K | 100(2) | 100(2) | 293(2) |
| λ, Å | 0.71073 | 0.71073 | 0.71073 |
| Crystal system | Monoclinic | Orthorhombic | Orthorhombic |
| Space Group | *P*2_1_ | *Pca*2_1_ | *Pna*2_1_ |
| a, Å | 12.4379(4) | 26.4059(19) | 13.4116(6) |
| b, Å | 13.5351(5) | 16.3678(13) | 20.7073(9) |
| c, Å | 19.3724(7) | 10.8345(9) | 18.0059(7) |
| α, ° | 90 | 90 | 90 |
| β, ° | 91.0170(10) | 90 | 90 |
| γ, ° | 90 | 90 | 90 |
| Cell Volume, Å^3^ | 3260.8(2) | 4682.7(6) | 5000.6(4) |
| Z | 2 | 4 | 4 |
| D_c_, g cm^-3^ | 1.481 | 1.441 | 1.564 |
| μ, mm^-1^ | 1.405 | 1.331 | 1.799 |
| F(000) | 1528 | 2120 | 2432 |
| Crystal size, mm | 0.16×0.14×0.11 | 0.12×0.06×0.05 | 0.24×0.19×0.15 |
| θ limits, ° | 15 | 1.542-25.050 | 1.499-25.997 |
| Index ranges | -44 ≤ h ≤ 15  -16 ≤ k ≤ 16  -23 ≤ l ≤ 23 | -31 ≤ h ≤ 31  -19 ≤ k ≤ 19  -12 ≤ l ≤ 12 | -16 ≤ h ≤ 16  -25 ≤ k ≤ 25  -22 ≤ l ≤ 22 |
| Reflections collected | 45686 | 54936 | 68925 |
| Independent reflections | 12808 [R_int_ = 0.0447] | 8064 [R_int_ = 0.2336] | 9743 [R_int_ = 0.0569] |
| Completeness to θ max | 100.0% | 98.5% | 99.5% |
| Data / restraints / parameters | 12808 / 7 / 769 | 8064 / 247 / 539 | 9743 / 408 / 651 |
| Goodness on fit on F^2^ | 1.065 | 1.297 | 1.070 |
| R_1_ (I > 2σ(I)) | 0.0219 | 0.1535 | 0.0381 |
| wR_2_ (all data) | 0.0548 | 0.2580 | 0.1020 |
| Largest diff. peak and hole, e Å^-3^ | 0.426 / -0.323 | 0.816 / -0.734 | 0.758 / -0.379 |

**REFERENCES**

1. M. Bortoluzzi, I. Ciabatti, C. Cesari, C. Femoni, M. C. Iapalucci, S. Zacchini, Synthesis of the Highly Reduced [Fe_6_C(CO)_15_]^4–^ Carbonyl Carbide Cluster and Its Reactions with H^+^ and [Au(PPh_3_)]^+^, *Eur. J. Inorg. Chem.* **2017**, 3135-3143.
2. C. F. Macrae, I. Sovago, S. J. Cottrell, P. T. A. Galek, P. McCabe, E. Pidcock, M. Platings, G. P. Shields, J. S. Stevens, M. Towler, P. A. Wood, Mercury 4.0: from visualization to analysis, design and prediction, *J. Appl. Crystallogr.* **2020**, *53*, 226−235.
3. S. Grimme, J. G. Brandenburg, C. Bannwarth, A. Hansen, Consistent Structures and Interactions by Density Functional Theory with Small Atomic Orbital Basis Sets, *J. Chem. Phys.* **2015**, *143*, 054107.
4. F. Weigend, R. Ahlrichs, Balanced Basis Sets of Split Valence, Triple Zeta Valence and Quadruple Zeta Valence Quality for H to Rn: Design and Assessment of Accuracy, *Phys. Chem. Chem. Phys.* **2005**, *7*, 3297.
5. V. Barone, M. Cossi, Quantum Calculation of Molecular Energies and Energy Gradients in Solution by a Conductor Solvent Model, *J. Phys. Chem. A* **1998**, *102*, 1995–2001.
6. J.-D. Chai, M. Head-Gordon, Long-Range Corrected Hybrid Density Functionals with Damped Atom–Atom Dispersion Corrections, *Phys. Chem. Chem. Phys.* **2008**, *10*, 6615.
7. T. Lu, F. Chen, Multiwfn: A Multifunctional Wavefunction Analyzer, *J. Comput. Chem.* **2012**, *33*, 580–592.
8. J. Contreras-García, E. R. Johnson, S. Keinan, R. Chaudret, J.-P. Piquemal, D. N. Beratan, W. Yang, NCIPLOT: A Program for Plotting Noncovalent Interaction Regions, *J. Chem. Theory Comput.* **2011**, *7*, 625–632.
9. C. Bannwarth, S. Ehlert, S. Grimme, GFN2-xTB—An Accurate and Broadly Parametrized Self-Consistent Tight-Binding Quantum Chemical Method with Multipole Electrostatics and Density-Dependent Dispersion Contributions, *J. Chem. Theory Comput.* **2019**, *15*, 1652–1671.
10. G. M. Sheldrick, SADABS-2008/1-Bruker AXS Area Detector Scaling and Absorption Correction; Bruker AXS: Madison, WI, 2008.
11. G. M. Sheldrick, Crystal Structure Refinement with SCHELXL, *Acta Crystallogr., Sect. C: Struct. Chem.* **2015**, *71*, 3−8.
12. T. Funaioli, C. Cesari, B. Berti, M. Bortoluzzi, C. Femoni, F. Forti, M. C. Iapalucci, G. Scorzoni, S. Zacchini, Chemical and Electrochemical Investigation of the Oxidation of a Highly Reduced Fe_6_C Iron Carbide Carbonyl Cluster: A Synthetic Route to Heteroleptic Fe_6_C and Fe_5_C Clusters, *Inorg. Chem.* **2025**, *64*, 9744-9757.
